# Maternal 5^m^CpG Imprints at the *PARD6G-AS1* and *GCSAML* Differentially Methylated Regions Are Decoupled From Parent-of-Origin Expression Effects in Multiple Human Tissues

**DOI:** 10.3389/fgene.2018.00036

**Published:** 2018-03-01

**Authors:** Graziela de Sá Machado Araújo, Ronaldo da Silva Francisco Junior, Cristina dos Santos Ferreira, Pedro Thyago Mozer Rodrigues, Douglas Terra Machado, Thais Louvain de Souza, Jozimara Teixeira de Souza, Cleiton Figueiredo Osorio da Silva, Antônio Francisco Alves da Silva, Claudia Caixeta Franco Andrade, Alan Tardin da Silva, Victor Ramos, Ana Beatriz Garcia, Filipe Brum Machado, Enrique Medina-Acosta

**Affiliations:** ^1^Núcleo de Diagnóstico e Investigação Molecular, Laboratório de Biotecnologia, Centro de Biociências e Biotecnologia, Universidade Estadual do Norte Fluminense Darcy Ribeiro, Campos dos Goytacazes, Brazil; ^2^Laboratório Nacional de Computação Científica, Petrópolis, Brazil; ^3^Faculdade de Medicina de Campos, Campos dos Goytacazes, Brazil; ^4^Faculdade Metropolitana São Carlos, Bom Jesus do Itabapoana, Brazil; ^5^Faculdade de Medicina de Ribeirão Preto, Universidade de São Paulo, Ribeirão Preto, Brazil

**Keywords:** *PARD6G-AS1*, *GCSAML*, *GRB10*, *ZNF124*, *OR2L13*, *LOC284240*, genomic imprinting, imprinting disease

## Abstract

A hallmark of imprinted genes in mammals is the occurrence of parent-of-origin-dependent asymmetry of DNA cytosine methylation (5^m^C) of alleles at CpG islands (CGIs) in their promoter regions. This 5^m^CpG asymmetry between the parental alleles creates allele-specific imprinted differentially methylated regions (iDMRs). iDMRs are often coupled to the transcriptional repression of the methylated allele and the activation of the unmethylated allele in a tissue-specific, developmental-stage-specific and/or isoform-specific fashion. iDMRs function as regulatory platforms, built through the recruitment of chemical modifications to histones to achieve differential, parent-of-origin-dependent chromatin segmentation states. Here, we used a comparative computational data mining approach to identify 125 novel constitutive candidate iDMRs that integrate the maximal number of allele-specific methylation region records overlapping CGIs in human methylomes. Twenty-nine candidate iDMRs display gametic 5^m^CpG asymmetry, and another 96 are candidate secondary iDMRs. We established the maternal origin of the 5^m^CpG imprints of one gametic (*PARD6G-AS1*) and one secondary (*GCSAML*) iDMRs. We also found a constitutively hemimethylated, nonimprinted domain at the *PWWP2AP1* promoter CGI with oocyte-derived methylation asymmetry. Given that the 5^m^CpG level at the iDMRs is not a sufficient criterion to predict active or silent locus states and that iDMRs can regulate genes from a distance of more than 1 Mb, we used RNA-Seq experiments from the Genotype-Tissue Expression project and public archives to assess the transcriptional expression profiles of SNPs across 4.6 Mb spans around the novel maternal iDMRs. We showed that *PARD6G-AS1* and *GCSAML* are expressed biallelically in multiple tissues. We found evidence of tissue-specific monoallelic expression of *ZNF124* and *OR2L13*, located 363 kb upstream and 419 kb downstream, respectively, of the *GCSAML* iDMR. We hypothesize that the *GCSAML* iDMR regulates the tissue-specific, monoallelic expression of *ZNF124* but not of *OR2L13*. We annotated the non-coding epigenomic marks in the two maternal iDMRs using data from the Roadmap Epigenomics project and showed that the *PARD6G-AS1* and *GCSAML* iDMRs achieve contrasting activation and repression chromatin segmentations. Lastly, we found that the maternal 5^m^CpG imprints are perturbed in several hematopoietic cancers. We conclude that the maternal 5^m^CpG imprints at *PARD6G-AS1* and *GCSAML* iDMRs are decoupled from parent-of-origin transcriptional expression effects in multiple tissues.

## Introduction

Genomic imprinting refers to the epigenetic and epigenomic differentiation of alleles of particular chromosomal loci on the basis of the parent transmitting them (Barlow and Bartolomei, 2014). The process of imprinting entails tagging with chemically stable marks consisting primarily of parent-of-origin-specific (and, therefore, asymmetrical) 5^m^CpG DNA methylation, enrichment with chemically modified histones, DNA accessibility, and the expression of long non-coding RNAs (lncRNAs) (Skaar et al., 2012). The trademark of genomic imprinting is the transcriptional silencing of the same parental allele in every cell (Chess, 2016). A hallmark of the canonical imprinted genes in mammals is the asymmetrical arrangement of parent-of-origin-dependent methylation at carbon 5 of the cytosine pyrimidine ring in the context of a CpG dinucleotide sequence (5^m^CpG) in CpG islands (CGIs) in the encompassed promoter regions (Yuen et al., 2011; Docherty et al., 2014; Hannula-Jouppi et al., 2014). The asymmetrical, parental-allele-specific status of 5^m^CpG at CGIs enables the construction of parent-of-origin-dependent regulatory platforms, which are built through the recruitment of chemical modifications to histones to achieve chromatin segmentations that impose differential repressive versus activation statuses on the parental alleles (Li and Zhang, 2014). Thus, the imprinting of the transmitted alleles from a subset of genes makes them subject to monoallelic transcriptional expression in specific tissues or developmental stages. Parent-of-origin-dependent allele-specific 5^m^CpG imprints are silencing epigenetic (e.g., heritable) marks that can be set in the germline or somatically acquired and conserved through mitotic divisions (Guo et al., 2014; Smith et al., 2014).

Genomic imprinting occurs in therian mammals (Barlow and Bartolomei, 2014) and angiosperms (Rodrigues and Zilberman, 2015). The exact repertoire of imprinted genes and the precise set of cis-acting regulatory elements, which make up the “imprintome” (Cooper and Constancia, 2010; Skaar et al., 2012), have not been established. In humans, approximately 150 chromosomal loci are known to be imprinted (Morison et al., 2005; Zhang et al., 2010; Jirtle and Murphy, 2012; Da Silva-Santiago et al., 2014; Wei et al., 2014; Cuellar Partida et al., 2017), although no unified, updated information repository is available. Through different imputation strategies, a predicted (candidate) imprinting status has been assigned to over 200 other loci (Luedi et al., 2007; Wei et al., 2014). Dysregulation of the epigenetic marks or mutations of the imprinted loci or domains is associated with 12 congenital diseases (Soellner et al., 2017).

Within the past 5 years, the reanalysis of big-data resource depositories of deep sequencing (methylomes, RNA-Seq and ChIP-Seq) from different tissues in a multitude of individuals or single cells at various developmental stages has substantially increased our ability to discover and characterize imprinted genes. The most informative epigenetic features currently used to detect imprinted loci are the following: (i) the occurrence of allelically methylated regions (AMRs) or genomic intervals exhibiting allele-specific (or allelically skewed) methylation (ASM) at CGIs (Fang et al., 2012; Song et al., 2013; Marzi et al., 2016); (ii) allele-specific expression (ASE) ratios across heterozygous SNPs (Babak et al., 2015; Baran et al., 2015); (iii) asymmetrical allele-specific enrichment of histone modifications associated with activation or repression states at the allele-specific candidate imprinted differentially methylated regions (iDMRs) (Savol et al., 2017); (iv) dysregulation of the intermediate methylation states at candidate iDMRs in diseased versus healthy tissues (Choufani et al., 2011; Docherty et al., 2014; Hannula-Jouppi et al., 2014; Maeda et al., 2014; Kagami et al., 2017); and, most recently, (v) cis methylation quantitative trait loci (cis-meQTLs) that exhibit parent-of-origin effects (POEs), with a physical distance ranging from 0.01 to 743 kb between the effector SNP and the affected 5^m^CpG site (Cuellar Partida et al., 2017).

Herein, through a comparative computational data mining approach, we report the identification of 125 novel candidate iDMRs in the human genome that integrated the maximal number of AMR records overlapping CGIs in methylomes from several tissues. We show that 29 candidate iDMRs display germline (gametic, primary) 5^m^CpG asymmetry, whereas 96 are candidate somatic (secondary) iDMRs. We revealed that the maternally inherited 5^m^CpG imprints for one gametic (*PARD6G-AS1*) and one somatic (*GCSAML*) iDMR are decoupled from POEs on the expression of the parental alleles. Lastly, we show that the oocyte-derived 5^m^CpG imprints are dysregulated in hematopoietic cancers.

## Materials and methods

### Subjects

Out of 100 trios from the Northern Region of the State of Rio de Janeiro, we included five nuclear families (mother, father, and son or daughter; median ages: 42, 50, and 19 years, respectively) whose genomic DNA (gDNA) had been genotyped as informative for the appropriate SNPs of the target genes. The investigators were blinded to the identities and scores of all trios throughout the genotyping process.

### DNA extraction

Human gDNA from freshly drawn peripheral blood samples (2 mL) was extracted using a commercial illustra blood genomicPrep Mini Spin Kit (GE Healthcare, Little Chalfont, UK), essentially as reported earlier (Machado et al., 2014). gDNA was quantified using an ND-NDL-PR NanoDrop Lite Spectrophotometer (Thermo Fisher Scientific, Waltham, MA, USA) and kept frozen at −20°C until used. For genotyping, aliquots of 10 ng of gDNA were used in each PCR reaction.

### Identification of candidate iDMRs

To identify novel candidate iDMRs, we employed an integrative data mining strategy based on the detection of AMRs, that is, chromosomal regions where the paternal allele is differentially methylated compared with the maternal allele (Fang et al., 2012; Song et al., 2013) and that overlap CGIs in whole-genome methylomes determined by bisulfite sequencing (BS-Seq) experiments. The strategy entailed extracting all fields (whole genome, hg19 assembly) from track data hubs (Raney et al., 2014) for CGIs that have any overlap with the coordinates of the AMR records reported in 39 BS-Seq methylome repositories having a bisulfite conversion rate of at least 0.95 and a minimum depth of 10 reads per CpG site (Dataset S1A: 39 BS-Seq methylomes). For this task, we used the UCSC Genome Browser Data Integrator web tool (Hinrichs et al., 2016), freely available at UCSC Genome Browser (Kent et al., 2002; Raney et al., 2014). We compared the integration results with the 15,282 AMRs identified previously in hg18 (Fang et al., 2012) by annotating them in hg19 using the UCSC Genome Browser LiftOver coordinate conversion tool. The output was used to populate a spreadsheet, and the data were filtered by the number of overlapping AMR records per CGI (Dataset S1B: Novel candidate iDMRs). As an investigative inclusion criterion, we accepted only CGIs that achieved at least 16 overlapping AMR records, which was the minimal number of overlapping records we observed for most of the known gametic (*n* = 20) and secondary (*n* = 9) iDMRs (Okae et al., 2014) (Dataset S1C: Known imprinted DMRs). The selected CGI-bearing AMRs were classified as candidate gametic or secondary iDMRs (Dataset S1B) on the basis of asymmetrical hypermethylated versus hypomethylated statuses in BS-Seq methylomes from human oocytes (Okae et al., 2014) (customized in the UCSC Genome Browser as described previously; Alves da Silva et al., 2016) and spermatozoa (Molaro et al., 2011).

### Determination of the parental origin of the 5^m^CpG methylation imprints

To validate the intermediate methylation statuses observed in the candidate iDMRs in public BS-Seq methylomes, we set up methylation-sensitive restriction enzyme PCR (MSRE-PCR) triplex assays (Dataset S1D: Primers and MSRE assay) specific to the candidate iDMRs; these assays were designed following the rationale described previously (Alves da Silva et al., 2016). Each test amplifies three loci: (i) An *Hpa*II-containing X-chromosome-specific monomorphic locus at the predicted promoter region of the *PPP2R3B* gene, located on the pseudoautosomal region PAR1 in females and the Y chromosome in males. The *PPP2R3B* promoter region is unmethylated in healthy and cancerous tissues from males and females (Figure S1); thus, it is not subject to gender-related differential methylation or effects of X-chromosome inactivation in women. *PPP2R3B* is a gonosomal melanoma tumor suppressor gene (van Kempen et al., 2016). The amplimer from this region was used as a positive control for the *Hpa*II restriction enzyme digestion. (ii) The candidate iDMR, encompassing at least one *Hpa*II recognition site and an informative SNP. (iii) A CpG-rich region of the *WRB* gene, bearing no *Hpa*II sites, used as a reference control for normalization of the ratio of restriction-enzyme-resistant 5^m^CpG sites in the target candidate iDMR. The methylation statuses were calculated as the proportion of restriction-enzyme-resistant 5^m^CpG sites using the equation reported previously (Alves da Silva et al., 2016). We genotyped the methylated alleles of informative SNPs (Dataset S1D), e.g., the alleles resistant to digestion with the restriction enzyme, using single-nucleotide primer extension (SNuPE) and SNaPshot technology (Thermo Fisher Scientific).

### DNA CpG methylation changes at candidate iDMRs in the placenta

Although there is widespread interindividual polymorphic methylation in the gametic maternal iDMRs that are specific to the human placenta (Hanna et al., 2016), variable maternal methylation in other tissues has been reported only in the somatic maternal iDMR of the tumor suppressor ncRNA *VTRNA2-1* (Paliwal et al., 2013; Romanelli et al., 2014; Green et al., 2015). Thus, we annotated the methylation statuses at the selected *PARD6G-AS1, PWWP2AP1*, and *GCSAML* iDMRs in 90 public methylome experiments for signs of polymorphic methylation. The inherent statuses are based on methylation profiling by the Illumina Infinium HumanMethylation450K BeadChip array (Hatt et al., 2015, 2016; Hanna et al., 2016). As a quality assurance control, we excluded from the analysis all unspecific cross-reactive probes, probes with *p* > 0.01, and probes varying among replicates (Chen et al., 2013). The placenta dataset comprises samples from healthy controls (*n* = 52); cases of preeclampsia (*n* = 8); and cases of trisomy 13 (Patau syndrome) (*n* = 6), 18 (Edwards syndrome) (*n* = 12) and 21 (Down syndrome) (*n* = 12) (Dataset S1E: Placenta GEO entries). We also included BS-Seq methylomes from three normal placentas (Dataset S1E). We customized tracks at the UCSC Genome Browser and extracted the 5^m^CpG levels to measure the mean values and standard deviation across the corresponding chromosomal regions.

### DNA CpG methylation changes at the candidate iDMRs in BS-Seq methylomes from cancer

Given that aberrant methylation associated with iDMRs is reported in cancer (Barrow et al., 2015), we annotated the variations in the methylation statuses of the candidate iDMRs exhibited in BS-Seq methylomes from hematopoietic cancers in the BLUEPRINT project (August 2016 data release) (Adams et al., 2012) (Dataset S1F: BLUEPRINT malignancies), compared with the intermediate methylation status typically observed in BS-Seq methylomes from healthy tissues. We restricted the analysis to hematopoietic cancers because the *GCSAML* candidate imprinted gene encodes a putative signaling protein associated with the sites of proliferation and differentiation of mature B lymphocytes (NCBI Resource Coordinators, 2017). The BLUEPRINT project was initially designed to decode the epigenetic signatures of healthy blood and hematopoietic malignancies. The project included samples from bone marrow (acute lymphocytic leukemia, acute myeloid leukemia, acute promyelocytic leukemia, and multiple myeloma) and venous blood (acute lymphocytic leukemia, acute myeloid leukemia, chronic lymphocytic leukemia, mantle cell lymphoma, and T-cell prolymphocytic leukemia). We viewed the target regions using the track hub available at the UCSC Genome Browser (Raney et al., 2014), with the hg38 chromosomal coordinates. We extracted the values of the 5^m^CpG calls, determined the average methylation level across the CpG sites that span the corresponding physical region in healthy tissues, and normalized the data by excluding the CpG sites with no data.

### Assessment of transcriptional allele-specific expression

For the genes within 2.3 Mb of the candidate iDMRs in either direction, we queried RNA-Seq experiments for signs of ASE across SNPs in dbSNP (Sherry et al., 2001) having global minor allele frequency (MAF) ≥ 0.1 as reported by the 1,000 Genome Project (Sudmant et al., 2015). We used both secondary (pre-processed) and primary (unprocessed) sources of RNA-Seq experiments.

### Secondary source of RNA-Seq experiments

The secondary source comprised the free track hubs for the Genotype-Tissue Expression (GTEx) project (data release V6, October 2015) (The GTEx Project, 2015), available from the UCSC Genome Browser and the dbGaP Data Browser. The GTEx track hubs, as designed and provided by the Lappalainen lab at the New York Genome Center, part of the GTEx Analysis Working Group (Castel et al., 2015), enable users to access the supporting tables. The GTEx track hubs report ASE measurement values at imputed heterozygous variants identified from transcriptome and genotype data collected from 51 primary tissues in RNA-Seq experiments (8,555 samples from 570 postmortem donors) (Dataset S2A: GTEx sample info V7). The ASE measurement values provided by GTEx refer to the median difference in the number of RNA-Seq read counts between the two alleles in an imputed heterozygous SNP across donors, with a depth ≥8 reads per site per donor. ASE is calculated as [0.5–Ref_allele_read count/(Ref_allele_read count + Alt_allele_read count)] (Castel et al., 2015). We used the UCSC Data Integrator web tool to extract the information about the physical coordinates' overlapping gene names; minimum, median, maximum, and quartiles (Q1 and Q3) for ASE values; number of donors; and read depth at SNPs present in the target genes. Because the ancestral allele of an SNP can occur in the context sequence of a different locus, we filtered out such SNPs from the downstream processing using a Short Match script in R-codes. We note that <5% of all SNP sites across the genome are excluded this way. The expression data were classified per gene locus using a script in R-codes that sorts the evidence into four mutually exclusive ASE categories. The ASE categories and their parameters were as follows: strictly monoallelic (minimal, maximal and Q1 ASE values = 0.5); consistent with monoallelic (minimal ASE values <0.5, Q1 and maximal ASE values ≥ 0.33); strictly biallelic (minimal, maximal and Q1 ASE values = 0), and consistent with biallelic (minimal ASE values <0.5, Q1 ASE values <0.33, and maximal ASE values > 0). The R script was validated using the corresponding data for a reference gene set consisting of the 40 known imprinted genes reported by the GTEx Analysis Working Group (Dataset S2B: 40 known imprinted genes) (Babak et al., 2015; Baran et al., 2015), also accessible from the GTEx portal. For the final expression profile designation (on a per-gene and per-tissue basis), we called only the genes with at least three non-discordant informative SNPs per tissue, and a depth ≥12 reads per SNP.

### Primary source of RNA-Seq experiments

The primary source consisted of 2,164 RNA-Seq experiments (Dataset S2C: 2,164 SRA experiment atlas), representing an atlas of 25 tissues, selected from the NCBI RNA sequence read archive (SRA) public data repositories. The unicity (e.g., the certainty of excluding mixed samples and disease tissues) of each SRA experiment was established by querying SNPs in *SNURF, H19*, and *PLAGL1*, known imprinted genes expressed monoallelically in multiple tissues (Dataset S1D) using the NCBI SRA search web tool, essentially as reported (Alves da Silva et al., 2016). The query sequence strings were 29 nt in length, and the alleles were represented by the International Union of Pure and Applied Chemistry (IUPAC) substitution codes. The unicity inclusion criteria were as follows: (i) ASE ≥ 0.42 (consistent with monoallelic expression), (ii) occurrence of allele flip (both alleles observed monoallelically in individual tissues) and (iii) absence of strictly biallelic patterns.

### Assessment of histone modification enrichment

To compile evidence about the likely involvement of the candidate iDMR in the regulation of imprinting, we examined the enrichment of histone modifications associated with activation or repression chromatin states, including promoter and enhancer functions and DNA accessibility (e.g., inter-nucleosome DNase-hypersensitive site distribution). We used the Grid Visualization web resource (available at the Roadmap Epigenomics Browser from the NIH Roadmap Epigenomics Mapping Consortium Bernstein et al., 2010; Roadmap Epigenomics Consortium et al., 2015) and the WashU Epigenome Browser (Zhou et al., 2011) to analyze uniformly processed datasets. We also annotated the non-coding genome in the candidate iDMRs by computationally exploring integrative large-scale functional- and comparative-genomics datasets on gene expression (RNA-Seq, cDNA mapping, active transcriptional start site (TSS) mapping, 5′-cap mapping) using the FANTOM ZENBU Browser (Severin et al., 2014). In summary, we analyzed secondary data from chromatin immunoprecipitation and DNA sequencing (ChIP-Seq) of 31 histone modification marks in 22 healthy human somatic tissues and cell lines (Dataset S1G: Roadmap Epigenomics samples). We extracted both the observed (Roadmap Epigenomics Consortium et al., 2015) and the imputed data points (Ernst and Kellis, 2015) for histone modifications across the candidate iDMRs from every tissue experiment and plotted them using the software GraphPad Prism. The chromosomal segmentation enrichment was compared at five constitutive oocyte-derived iDMRs (*GRB10, INPP5F, ZNF331, KCNQ1, MEST*) and three secondary maternal iDMRs (*MAGEL2, NDN, MEG8*) (Dataset S1H: iDMR reference set). The *GRB10* iDMR was included as it regulates monoallelic expression from the paternal or maternal allele in a tissue- and isoform-specific fashion (Blagitko et al., 2000; Yoshihashi et al., 2000; Hitchins et al., 2001; McCann et al., 2001; Mergenthaler et al., 2001; Monk et al., 2009).

### Genotype-phenotype associations

We queried the PheGen*I* (Ramos et al., 2014), e-GRASP (Karim et al., 2016), PhenoScanner (Staley et al., 2016), and HaploReg v4.1 (Ward and Kellis, 2016) web database tools to cross-reference SNPs at the *PARD6G-AS1, PWWP2AP1*, and *GCSAML* candidate iDMRs (Dataset S3A: iDMR SNP lookup) with a broad range of phenotypes from large-scale genome-wide association studies (GWAS) to gain insights into possible disease-associated loci and to assign chromatin states to the lead variants. We set the *p* and r^2^ cut-off values at 5 × 10^−8^ and 0.8, respectively, and requested results for 1,000 Genomes SNP proxies at significant gametic disequilibrium. We ran the SNP variants within the *GRB10* iDMR (Dataset S3A) as a control set of SNPs that did not cross-reference with disease phenotypes in the above databases.

## Results

### Novel candidate iDMRs

**Figure 1:**
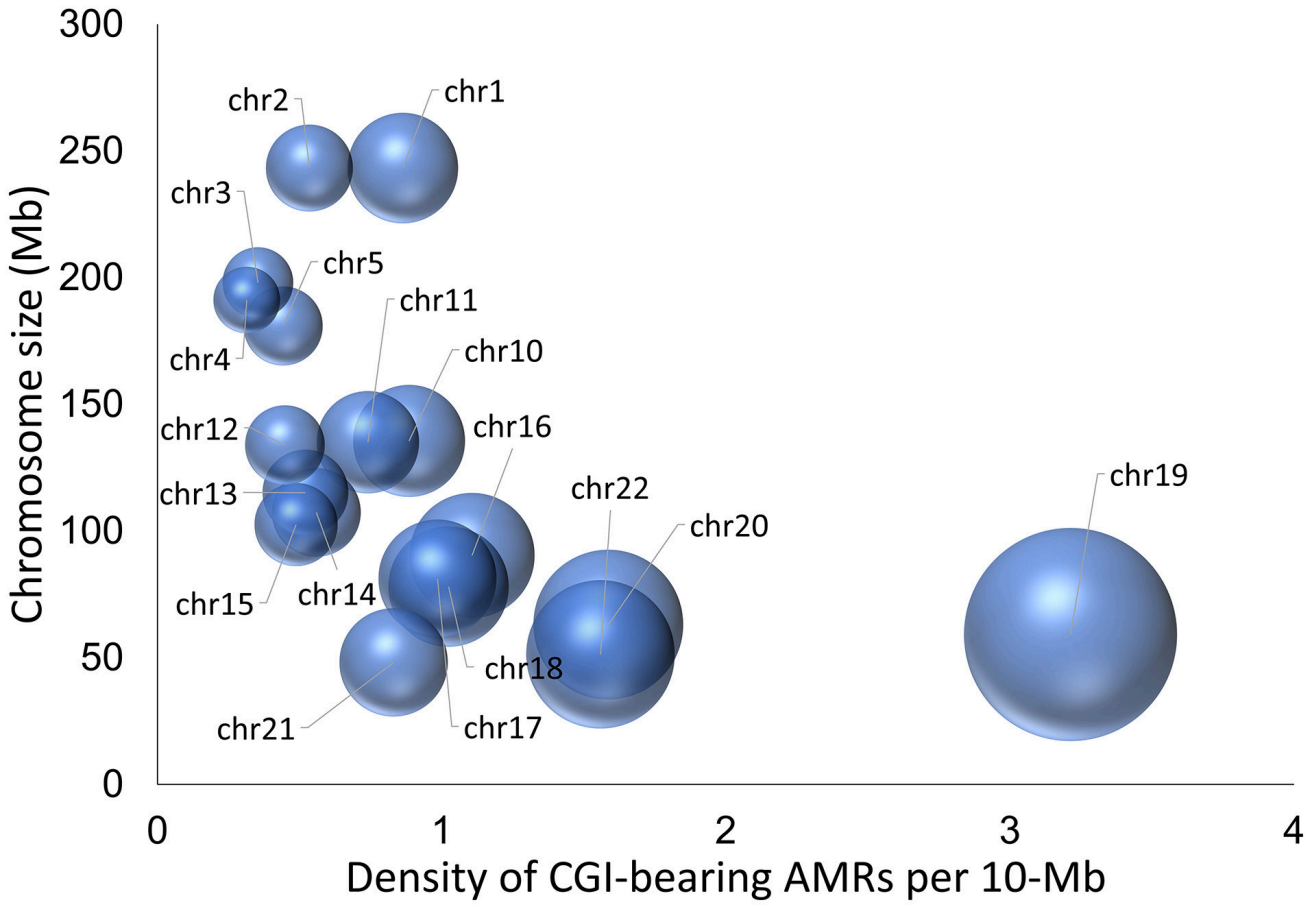
Three-dimensional bubble chart display of the density of novel candidate iDMRs. The diameter of the bubble represents the density of candidate iDMRs per 10 Mb autosomal region, whereas the position of the balloon on the *y*-axis is related to the chromosomal size in Mb. The highest density of candidate iDMRs is 3.1/Mb on chromosome 19.

To identify novel candidate iDMRs, we integrated AMR records that overlap annotated CGIs from 39 BS-Seq methylomes, including 26 primary (uncultured) healthy tissues and 13 cell types and cell lines (Dataset S1A). We limited the analysis to the autosomes because evidence of 5^m^CpG imprinted marks on the X chromosome in women is only now beginning to emerge. We further restricted the screening to CGIs overlapping AMR records in at least 16 methylomes, which was the minimal number of records observed in 29 out of 67 known iDMRs (Okae et al., 2014; Dataset S1C). The analysis yielded 480 CGIs, 168 of which consistently exhibited methylation levels ranging from 0.35 to 0.65 at the overlapping CpG sites in the esophagus tissue methylome, as the reference methylome because every chosen CGI has at least one AMR record in that tissue methylome (Dataset S1B). The distribution of the 168 CGI-bearing AMRs per autosome is shown in Figure 1. Chromosomes 6 through 9 had no hits. Chromosome 19 bears the highest density of hits per megabase. Eleven CGI-bearing AMRs correspond to known gametic iDMRs (10 maternal and one paternal), six to known secondary iDMRs (Okae et al., 2014), and 26 to known candidate iDMRs for which parent-of-origin-specific methylation is uncertain (Court et al., 2013, 2014; Docherty et al., 2014) (Dataset S1B). The 125 remaining CGI-bearing AMRs appear to be new candidate iDMRs. Of these, 29 exhibit asymmetrical methylation statuses in the methylomes of oocytes and spermatozoa; thus, they were classified as candidate gametic iDMRs (18 maternal and 11 paternal). Ninety-six are candidate secondary iDMRs (Dataset S1B).

**Figure 2:**
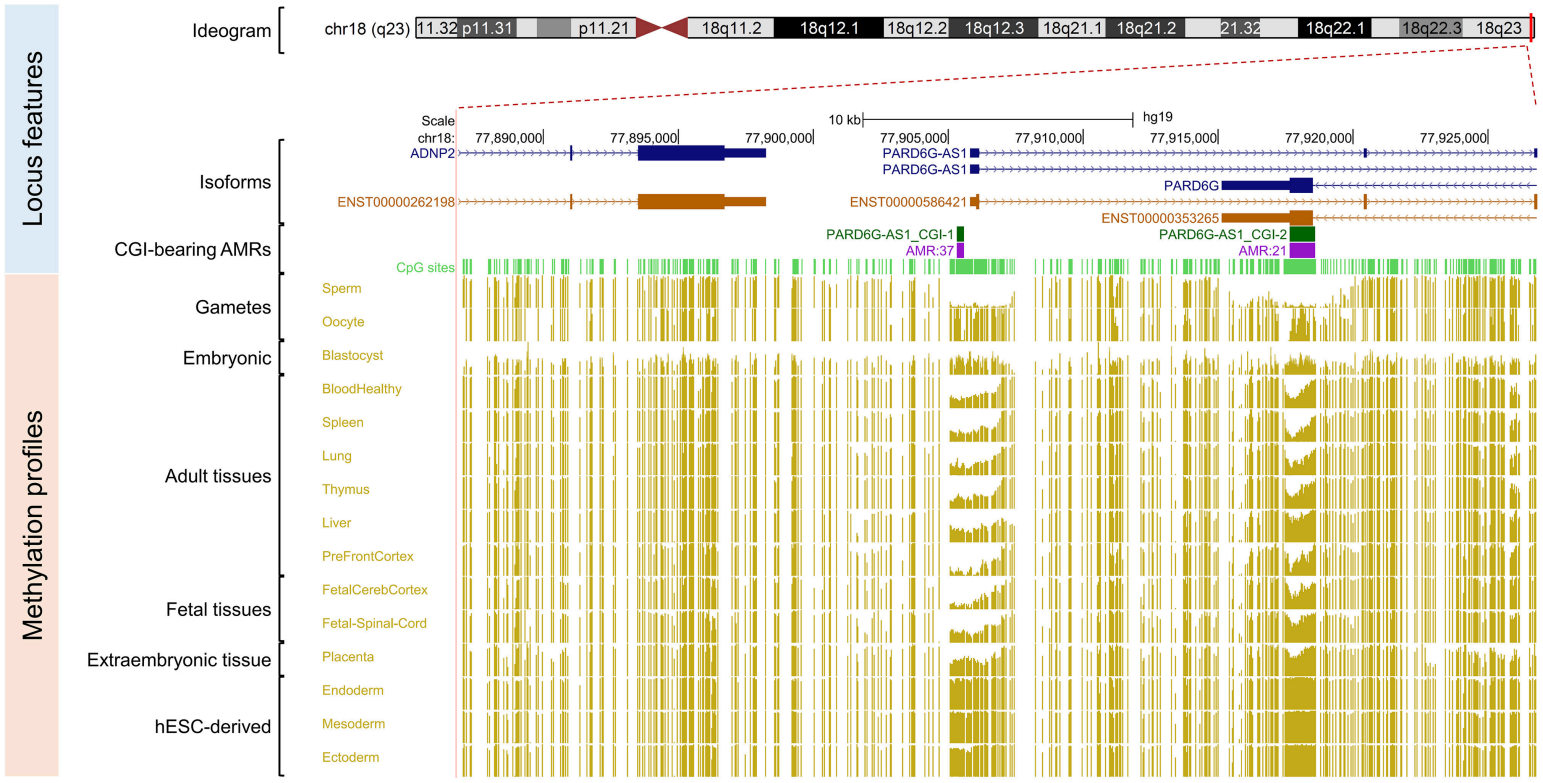
Primary intermediate methylation status at the *PARD6G-AS1* predicted promoter region. Chromosome 18 ideogram; physical positions and domain features of the *PARD6G-AS1* locus depicting the methylation status at the CpG sites (golden ticks) across a 40 kb long-span view (hg19; chr18:77886816–77926815). The methylation levels are represented on a scale from 0 to 1 (hypomethylated to hypermethylated). The image is centered on the CGI localized in the *PARD6G-AS1* predicted promoter region, labeled “*PARD6G-AS1* CGI-1.” The light green ticks represent the positions of the CpG sites. The methylation level across the *PARD6G-AS1* CGI ranges from 0.35 to 0.65 in adult somatic tissues. In the same region, there is asymmetrical methylation in gametes (hypermethylation in oocytes and hypomethylation in spermatozoa). The *PARD6G-AS1* gene partly overlaps the downstream *PARD6G* gene locus, which is transcribed in the opposite direction from the minus DNA strand. The annotated features are (from top to bottom) the exon-intron organization of the principal and alternative *PARD6G-AS1* and *PARD6G* splice isoforms (variants 1 and 2 in shades of dark blue) and the species-conserved *PARD6G-AS1* and *PARD6G* principal isoform transcripts (ENST00000586421 and ENST00000353265, respectively, in brown) (Rodriguez et al., 2013).

**Figure 3:**
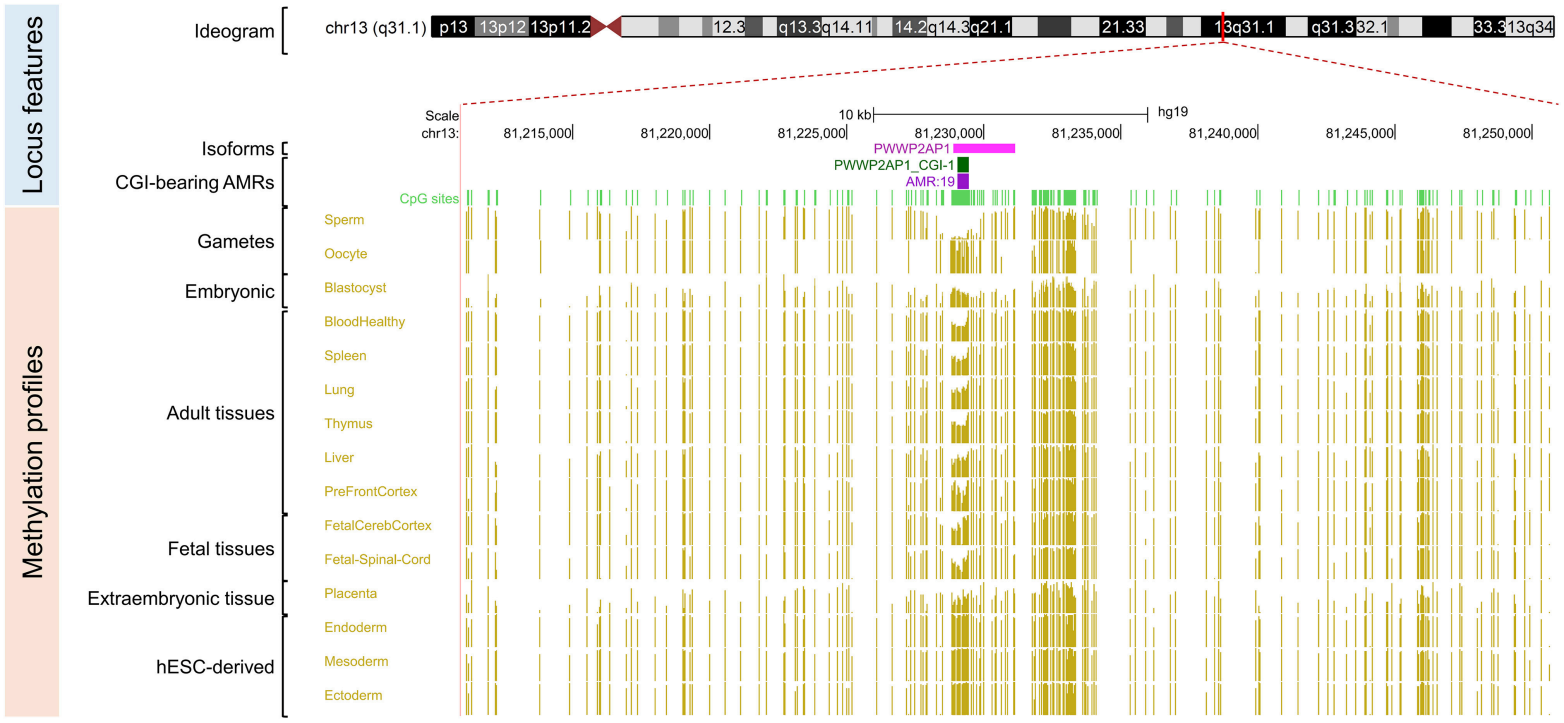
Primary intermediate methylation status at the *PWWP2AP1* processed pseudogene. Chromosome 13 ideogram; physical positions and domain features of the *PWWP2AP1* locus showing the methylation status at the CpG sites (golden ticks) across a 40 kb long-span view (hg19; chr13:81210960-81250959). The methylation levels are represented on a scale from 0 to 1 (hypomethylated to hypermethylated). The image is centered on the CGI localized in the *PWWP2AP1* predicted promoter-flanking region, labeled “*PWWP2AP1* CGI-1.” The light green ticks represent the positions of the CpG sites. The methylation across the CpG sites within the *PWWP2AP1* candidate iDMR ranges from 0.35 to 0.65 in adult somatic tissues. In the same region, however, there is asymmetrical methylation in gametes (hypermethylation in oocytes and hypomethylation in spermatozoa). The GENCODE ENST00000429776.2 transcript for the *PWWP2AP1* locus is shown. The annotated features (from top to bottom) are as in Figure 2.

**Figure 4:**
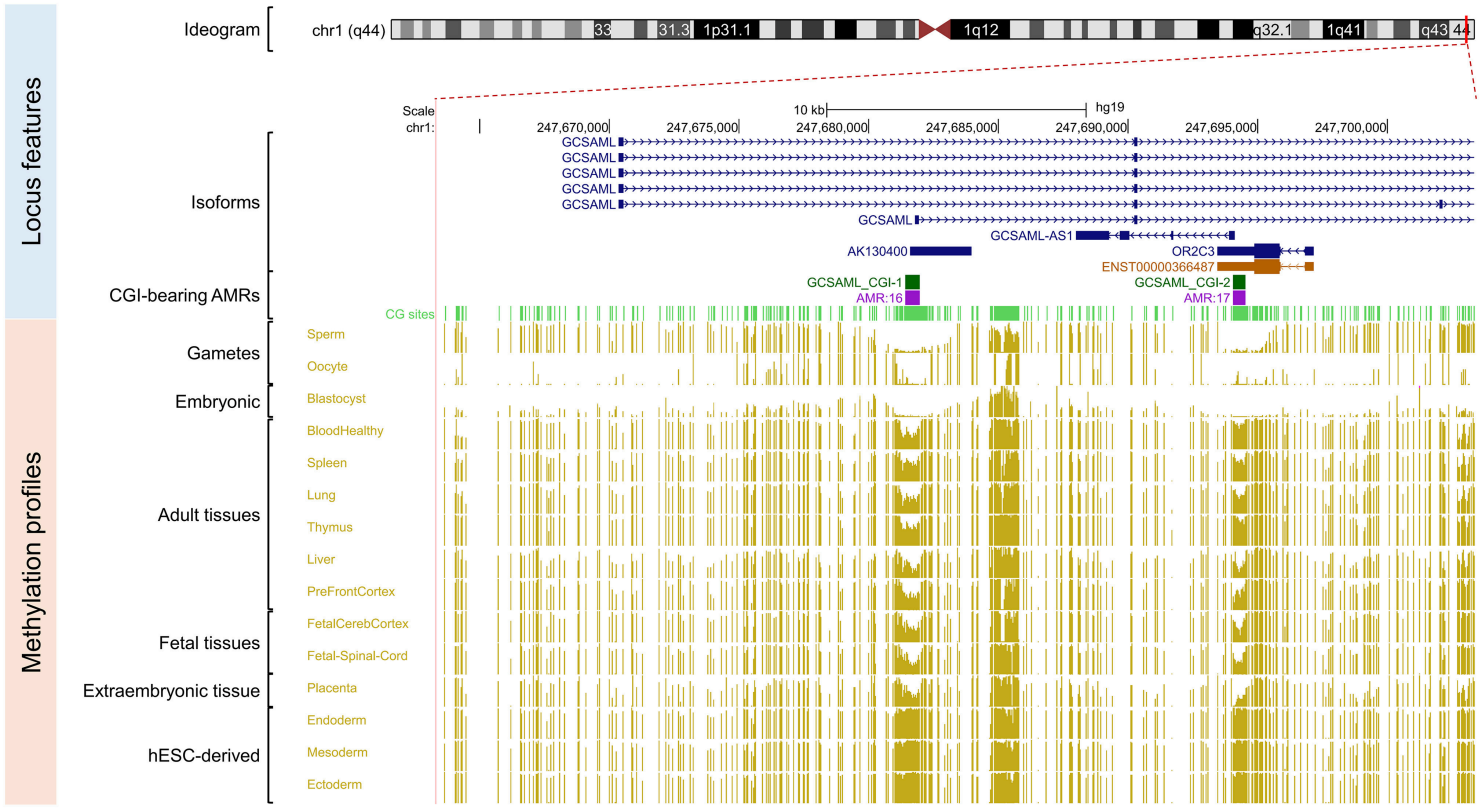
Secondary intermediate methylation status at the *GCSAML* predicted secondary promoter. Chromosome 1 ideogram; physical positions and domain features of the *GCSAML* locus displaying the methylation status at the CpG sites (golden ticks) across a 40 kb long-span view (hg19; chr1:247663350-247703349). The methylation levels are represented on a scale from 0 to 1 (hypomethylated to hypermethylated). The image is centered on the intronic CGI (*GCSAML* CGI-1 track) near the *GCSAML* predicted secondary promoter-flanking region. The light green ticks represent the positions of the CpG sites. The *GCSAML* locus overlaps two other annotated genes (*GCSAML-AS1* and *OR2C3*). The annotated features (from top to bottom) are as in Figure 2. The methylation level across the CpG sites within the *GCSAML* candidate iDMR ranges from 0.35 to 0.65 in adult somatic tissues. In methylomes from male and female gametes and from blastocysts, this region is hypomethylated.

**Figure 5:**
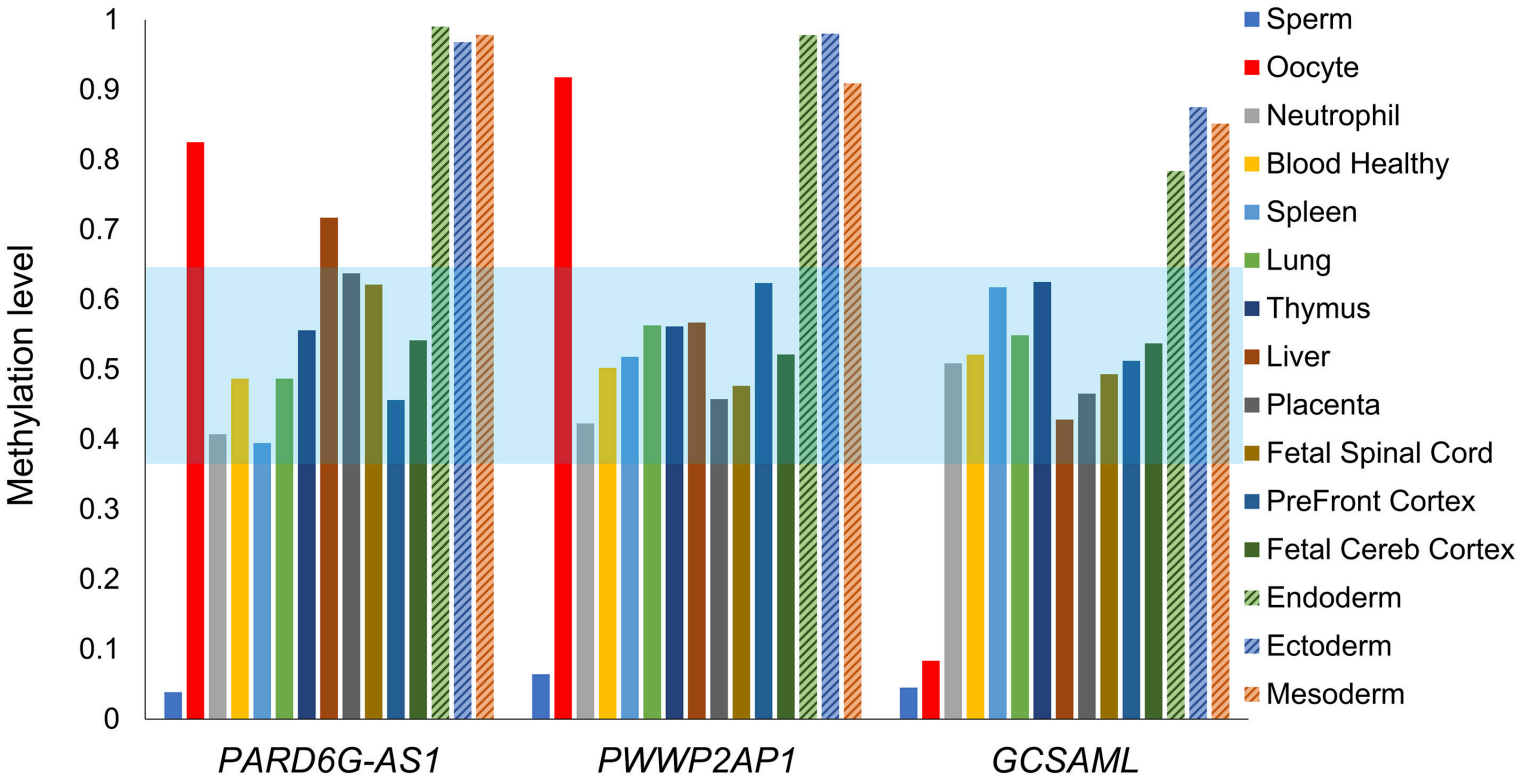
Intermediate mean methylation level across the *PARD6G-AS1, PWWP2AP1*, and *GCSAML* candidate iDMRs. Comparative variation in the average methylation levels across all 5^m^CpG sites on the candidate iDMRs in the methylomes of healthy tissues depicted in Figures 2–4. In adult somatic tissues, the overall mean levels were 0.39 (*PARD6G-AS1* iDMR), 0.42 (*PWWP2AP1* iDMR) and 0.42 (*GCSAML* iDMR). A slightly skewed hypermethylation state (0.70) was observed in the liver for the *PARD6G-AS1* iDMR. The three candidate iDMR regions are consistently hypermethylated in hESC-derived CD56^+^ ectoderm and mesoderm and hESC-derived CD184^+^ endoderm cell cultures. The light blue zone represents the expected intermediate methylation range (0.35–0.65). Each methylome is represented by a different color of bar.

Because our laboratory is interested in the discovery of imprinted genes located in the autosomes that are commonly affected by nondisjunction (Alves da Silva et al., 2016), we selected two candidate gametic maternal iDMRs—*PARD6G-AS1* (par-6 family cell polarity regulator gamma antisense RNA 1 on chromosome 18; Edwards syndrome) (Figure 2) and *PWWP2AP1* (PWWP domain containing 2A pseudogene 1 on chromosome 13; Patau syndrome) (Figure 3)—and the secondary candidate iDMR in the *GCSAML* (germinal center associated signaling and motility like) gene on chromosome 1 (Figure 4), the autosome with the highest number of candidate iDMR hits (Figure 1). The three candidate iDMRs consistently exhibited intermediate methylation profiles in somatic tissues (levels ranging from 0.35 to 0.65 across all CpG sites) but were hypermethylated (>0.65) in human embryonic stem cell (hESC)-derived CD56^+^ ectoderm and mesoderm and CD184^+^ endoderm cell cultures (Figure 5).

We validated the intermediate methylation profiles observed in public methylomes in the selected candidate iDMRs using gDNA from venous blood by DMR-specific MSRE-PCR triplex assays (Figure S2).

**Figure 6:**
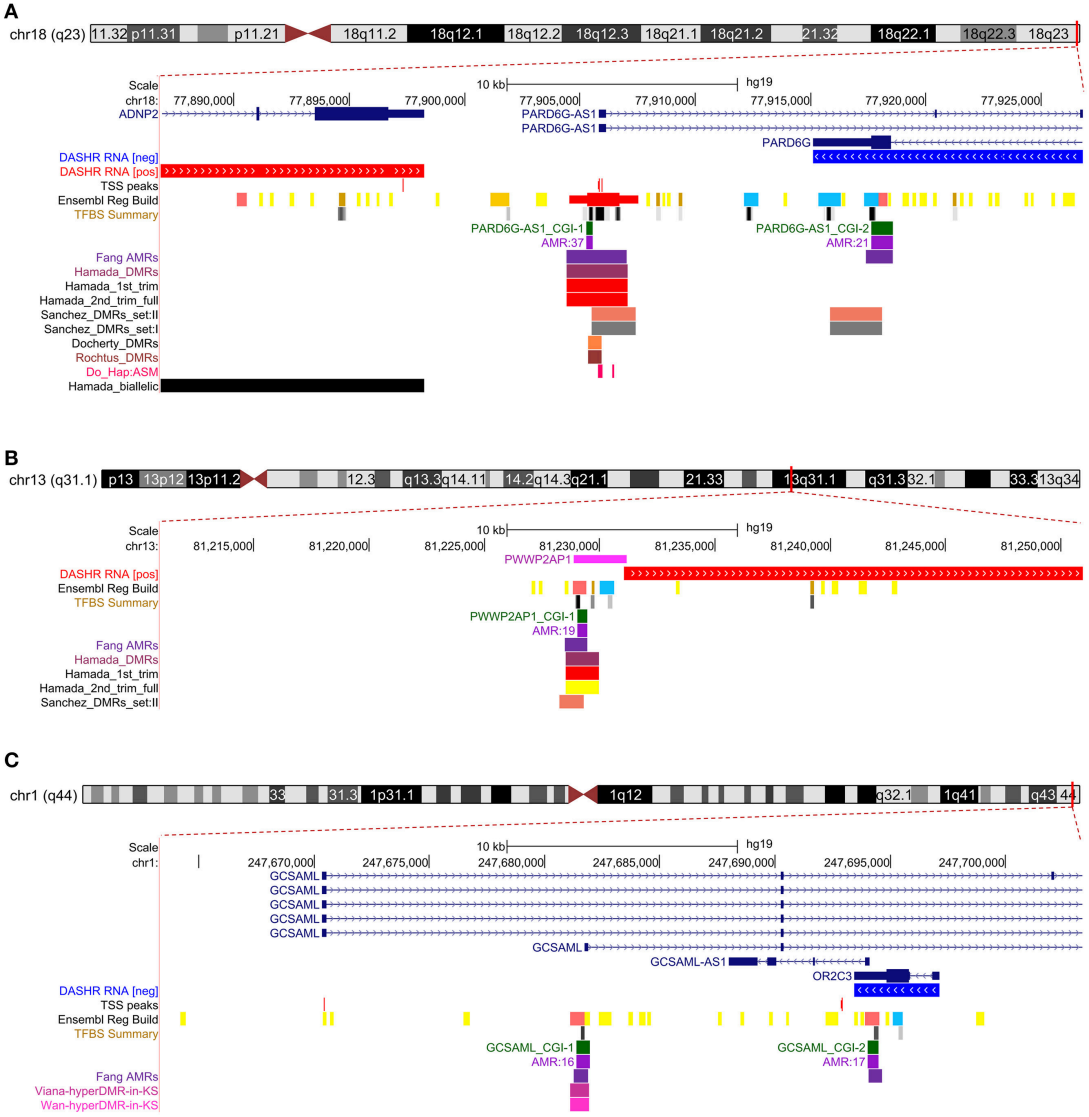
Cross-reference overlaps of the *PARD6G-AS1, PWWP2AP1*, and *GCSAML* candidate iDMRs with differentially methylated regions reported in control-disease methylomes and healthy placentas. Customized graphics of the intersections between the candidate iDMRs at *PARD6G-AS1* **(A)**, *PWWP2AP1* **(B)** and *GCSAML* **(C)** and the public records reporting parent-of-origin predictions, abnormal methylation profiles, and haplotype-dependent allele-specific 5^m^CpG sites. In each panel, the annotated features are (from top to bottom) the exon-intron organization of the principal and alternative isoforms, the evidence of large intergenic non-coding RNAs (lincRNAs) and small non-coding RNAs (sncRNAs) (DASHR RNA [pos] and [neg] tracks), the FANTOM5 transcriptional start sites (TSS peaks track) (Lizio et al., 2017), the Ensembl Regulatory Build predicted promoters (Ensembl Reg Build track) and transcriptional factor binding sites (TFBS summary track) (Cunningham et al., 2015), CpG islands and CGI-bearing AMRs (this study) and the comparative custom Fang_AMR track (Fang et al., 2012). **(A)** The *PARD6G-AS1* CGI-1 overlaps the domain previously predicted to be differentially methylated in a parent-of-origin-dependent manner in blastocysts and placenta (Okae et al., 2014; Hamada et al., 2016; Sanchez-Delgado et al., 2016a) (Hamada_DMRs, Sanchez_DMRs_set:I, and Sanchez_DMRs_set:II tracks, respectively), for which the parental origin of the methylated alleles had not been established. The allelic methylation levels in the aforementioned regions are maternally skewed, varying from 0.69 in first- and second-trimester to 0.82 in full-term placentas (Hamada_1st_trim and Hamada_2nd_trim_full tracks). The *PARD6G-AS1* CGI-1 also intersects with the domains reported to be hypomethylated in patients with Beckwith-Wiedemann syndrome and transient neonatal diabetes (Docherty et al., 2014) (Docherty_DMRs track) and in pseudohypoparathyroidism patients with *GNAS* cluster imprinting defects (Rochtus et al., 2016) (Rochtus_DMRs track), as well as with the haplotype-dependent allele-specific 5^m^CpG sites reported in placenta, liver, lung, brain and T cells (Do_Hap:ASM track) (Do et al., 2016). The genes that are known to be biallelically expressed in placenta (Hamada et al., 2016) are depicted in the Hamada_biallelic track. (B) The *PWWP2AP1* CGI-1 overlaps the domain previously predicted to be differentially methylated in a parent-of-origin-dependent manner in blastocysts and placenta (Hamada et al., 2016; Sanchez-Delgado et al., 2016a) (Hamada_DMRs, and Sanchez_DMRs_set:II tracks, respectively), for which the parental origin of the methylated alleles had not been established. The methylation levels in the aforementioned regions varied from 0.38 in first- and second-trimester placentas to 0.24 in full-term placentas (Hamada_1st_trim and Hamada_2nd_trim_full tracks). **(C)** The *GCSAML* CGI-1 overlaps the domain reported to be skewed toward hypermethylation in Klinefelter syndrome (47,XXY karyotype) versus control males (Viana et al., 2014; Wan et al., 2015) (Viana-hyperDMR-in-KS and Wan-hyperDMR-in-KS), for which the parental origin of the allele methylation had not been established.

We next cross-referenced the physical coordinates of the candidate iDMRs with the overlapping domains in public methylomes of control versus diseased specimens. We found that all three candidate iDMRs had been predicted to be differentially methylated in a maternally dependent manner in the placenta (Hamada et al., 2016; Sanchez-Delgado et al., 2016a), but the parental origin of the methylated alleles had not been established (Figures 6A–C). We observed that the *PARD6G-AS1* DMR domain is hypomethylated in three imprinted diseases—Beckwith-Wiedemann syndrome, transient neonatal diabetes (Docherty et al., 2014), and pseudohypoparathyroidism in patients with *GNAS* cluster imprinting defects (Rochtus et al., 2016)—compared with matched control subjects (Figure 6A). Moreover, we revealed that the *GCSAML* DMR is differentially hypermethylated in patients with Klinefelter syndrome (47,XXY) compared with matched males but not females (Viana et al., 2014; Wan et al., 2015; Figure 6C).

We also searched for evidence of haplotype-dependent allele-specific 5^m^CpG sites within the selected candidate iDMRs by integrating the corresponding records reported in methylomes (Do et al., 2016). Within the *PARD6G-AS1* candidate iDMR, we noted evidence of haplotype-dependent allele-specific 5^m^CpG sites in placenta, liver, lung, brain and T cells methylomes (Figure 6A).

### The 5^m^CpG sites at the *PARD6G-AS1* and *GCSAML* DMRs are maternally inherited

**Figure 7:**
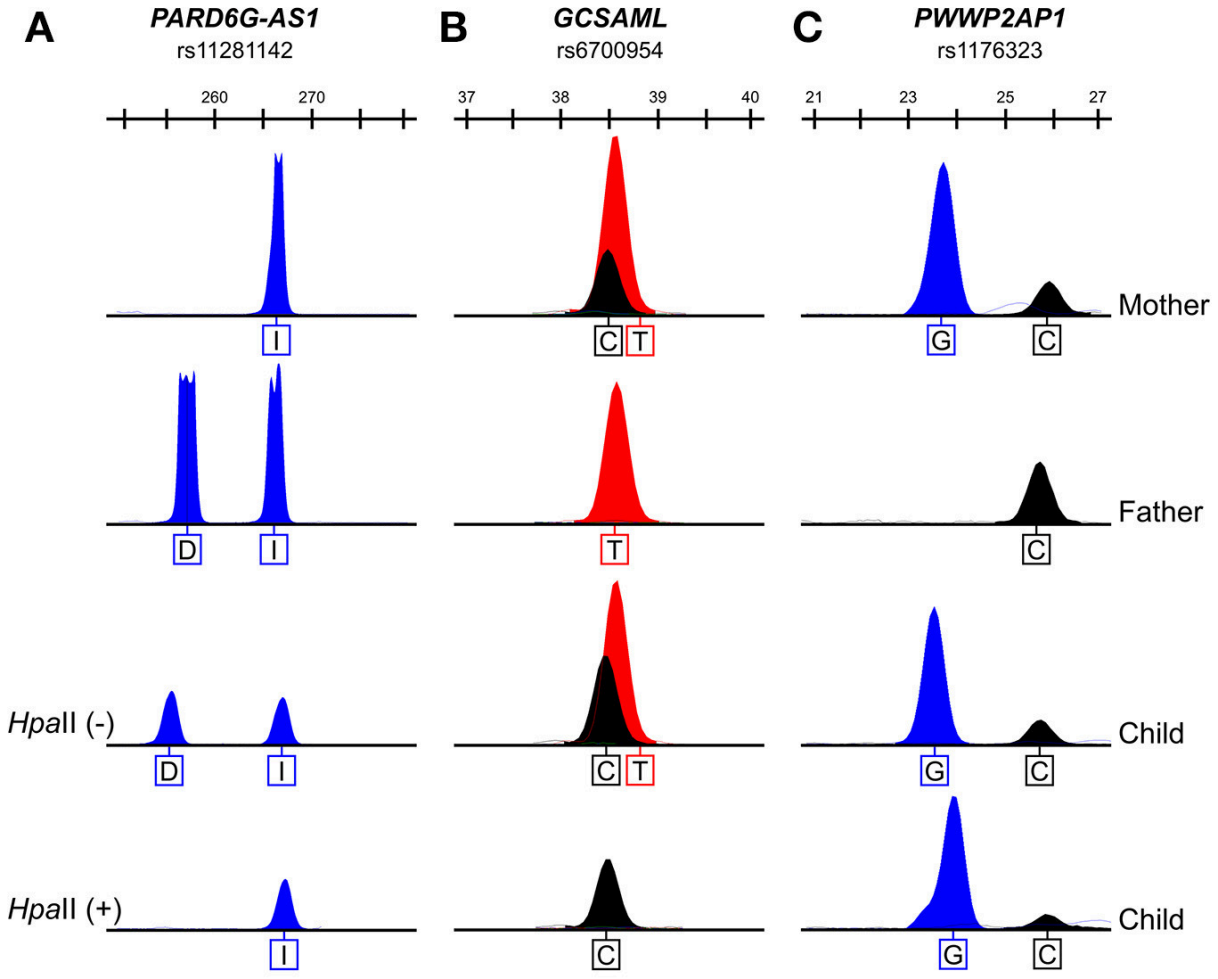
The 5^m^CpG sites at the *PARD6G-AS1* and *GCSAML* iDMRs are on the maternally transmitted alleles. The maternally transmitted alleles and not the paternally transmitted alleles at the *PARD6G-AS1* **(A)** and *GCSAML* **(B)** DMRs are consistently methylated, as supported by the resistance of the maternally inherited alleles to digestion with the methylation-sensitive *Hpa*II restriction enzyme and the lack of amplification of the paternally transmitted alleles from the digested genomic DNA samples for the informative variant SNPs (the *PARD6G-AS1* upstream gene indel variant rs11281142 (I = insertion of CTGTGGTGC; D = deletion of CTGTGGTGC) and the *GCSAML* intron variant/upstream gene variant rs6700954, respectively) in three representative nuclear families that were informative for at least one SNP. Therefore, the 5^m^CpG marks in the *PARD6G-AS1* and *GCSAML* DMRs are maternal imprints. On the other hand, both parental alleles of the *PWWP2AP1* rs1176323 variant are resistant to digestion **(C)**. Thus, the 5^m^CpG marks in the *PWWP2AP1* CGI-1 occur in a parent-of-origin-independent manner. Given the intermediate rate of methylation in *PWWP2AP1* CGI-1 (see Figure 5), the occurrence of 5^m^CpG sites in both parental alleles indicates that the *PWWP2AP1* CGI-1 alleles are methylated in a stochastic fashion.

The methylation statuses of the transmitted alleles were determined by genotyping the methylation-sensitive *Hpa*II-resistant alleles amplified from venous blood gDNA samples from nuclear trios (mother, father, and son or daughter) informative for SNPs within the candidate iDMRs for the *PARD6G-AS1* (upstream gene indel variant rs11281142: -/CTGTGGTGC), *PWWP2AP1* (rs1176323), and *GCSAML* (intron variant, upstream gene variant rs6700954) loci (Dataset S1D). The 5^m^CpG sites at the *PARD6G-AS1* (Figure 7A) and *GCSAML* (Figure 7B) DMRs are maternally inherited. By contrast, at the *PWWP2AP1* candidate iDMR, both parental alleles are methylated (Figure 7C). Given the asymmetrical methylation statuses observed in the BS-Seq methylomes of oocytes and spermatozoa at the *PARD6G-AS1* iDMR (Figure 2), we inferred that the 5^m^CpG imprints are primary (e.g., oocyte-derived). Thus, we classified this domain as a gametic maternal iDMR. On the other hand, the 5^m^CpG imprints at the *GCSAML* iDMR are not oocyte-derived but somatically acquired) (Figure 4), making the domain a secondary maternal iDMR. The primary versus secondary designations above are concordant with the emerging view that, in blastocysts, the primary iDMRs (Figures 2, 4) exhibit intermediate methylation levels while the secondary iDMRs are hypomethylated (Okae et al., 2014; Hamada et al., 2016; Sanchez-Delgado et al., 2016a).

The parent-of-origin-independent methylation at the *PWWP2AP1* candidate iDMR is not consistent with imprinting, despite the occurrence of the asymmetrical methylation observed in gametes (Figure 3). The biallelic methylation and the intermediate methylation seen at the *PWWP2AP1* DMR in various BS-Seq somatic methylomes, including those of blastocysts (Figure 3), support the scenario of random (e.g., switchable) allelic methylation of this domain. Therefore, the gametic 5^m^CpG asymmetry observed at the *PWWP2AP1* promoter CGI is not maintained in the somatic tissues investigated. The *PWWP2AP1* CGI appears to be the first example of a constitutively hemimethylated, nonimprinted domain that is hemimethylated in blastocysts.

### The maternal 5^m^CpG sites are not polymorphic in the placenta

**Figure 8:**
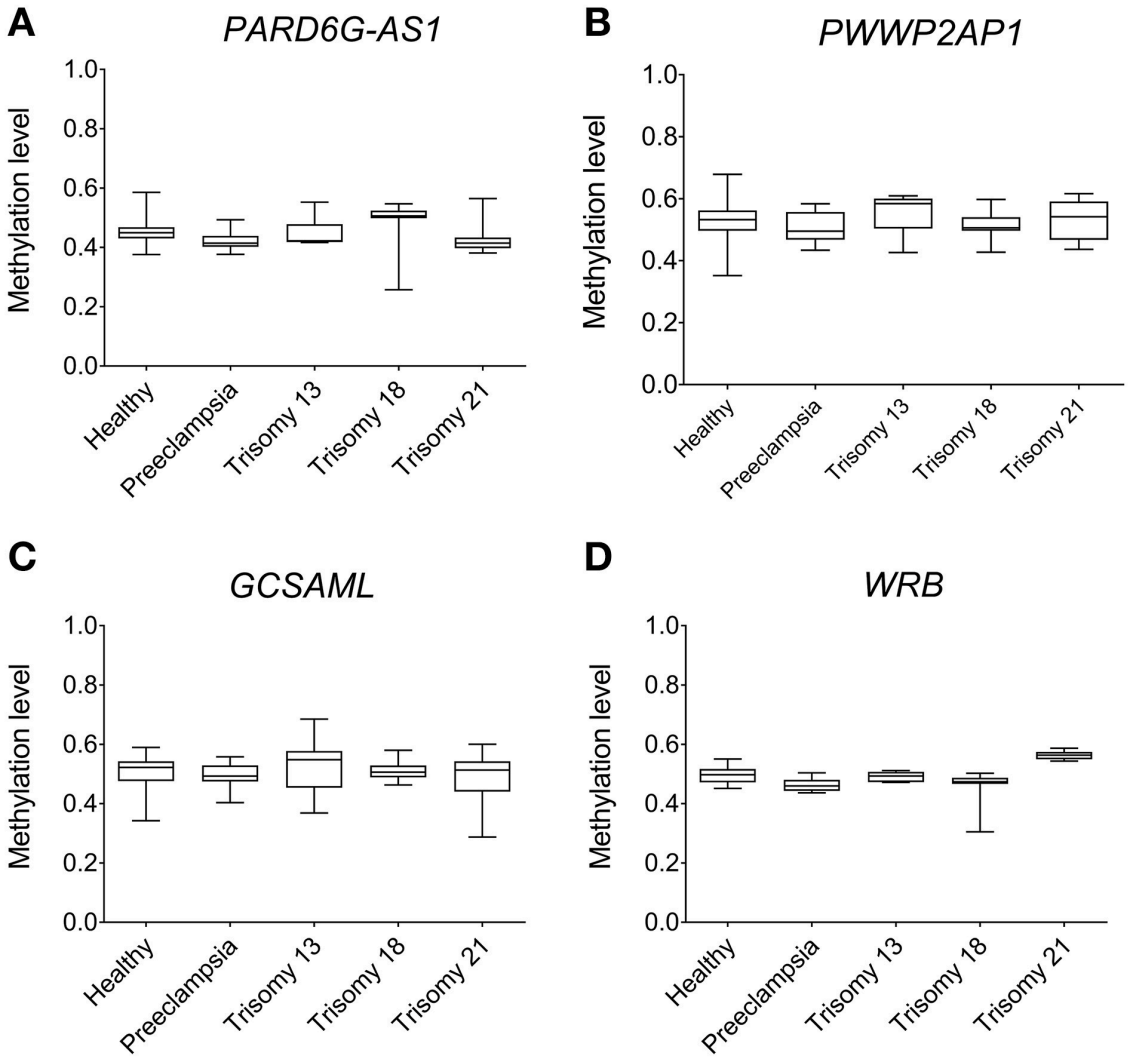
Absence of epipolymorphism at the *PARD6G-AS1* and *GCSAML* maternal iDMRs in placentas from healthy and diseased pregnancies. The new maternal iDMRs are consistently hemimethylated in 450 K array (*n* = 90) methylomes from healthy; preeclampsia; and trisomy 13, 18, and 21 placentas. Comparison of the observed methylation levels in **(A)** the *PARD6G-AS1* iDMR, **(B)** the *PWWP2AP1* DMR, **(C)** the *GCSAML* iDMR, and **(D)** the *WRB* iDMR. Note that none of the conditions led to an unmethylated or hypermethylated status at the iDMRs. The hypomethylated status at the *PARD6G-AS1* iDMR in some but not all trisomy 18 placentas suggests a paternal origin of the nondisjunction of the extra copy of chr18 in some placentas. The supernumerary chromosome 18 also perturbs the methylation status of the *WRB* iDMR in chromosome 21.

The methylation statuses at the *PARD6G-AS1* and *GCSAML* iDMRs in BS-Seq and 450K array placenta methylomes were coherent with the intermediate statuses observed in methylomes from diverse tissues (Figure 8). Overall, the intermediate methylation level was undisturbed in normal, preeclampsia, and trisomy 13, 18, and 21 placentas. However, we noted hypomethylation (observed lower limit 0.26) for the *PARD6G-AS1* iDMR in some samples from trisomy 18 (Figure 8A). The hypomethylated status of some but not all trisomy 18 placentas suggests a paternal origin of the nondisjunction of the supernumerary chromosome 18 in some placentas (e.g., two unmethylated paternal allele copies versus one maternal methylated allele; expected methylation rate ≤ 0.33). We reported a similar nondisjunction parent-of-origin effect for the known maternal gametic iDMR in the *WRB* gene in chromosome 21 in cases of paternal trisomy 21 (Alves da Silva et al., 2016). Interestingly, the methylation level in the *WRB* iDMR appears to be perturbed in trisomy 18 placentas (Figure 8D).

### 5^m^CpG DNA methylation dysregulation in the novel iDMRs in hematopoietic cancers

**Figure 9:**
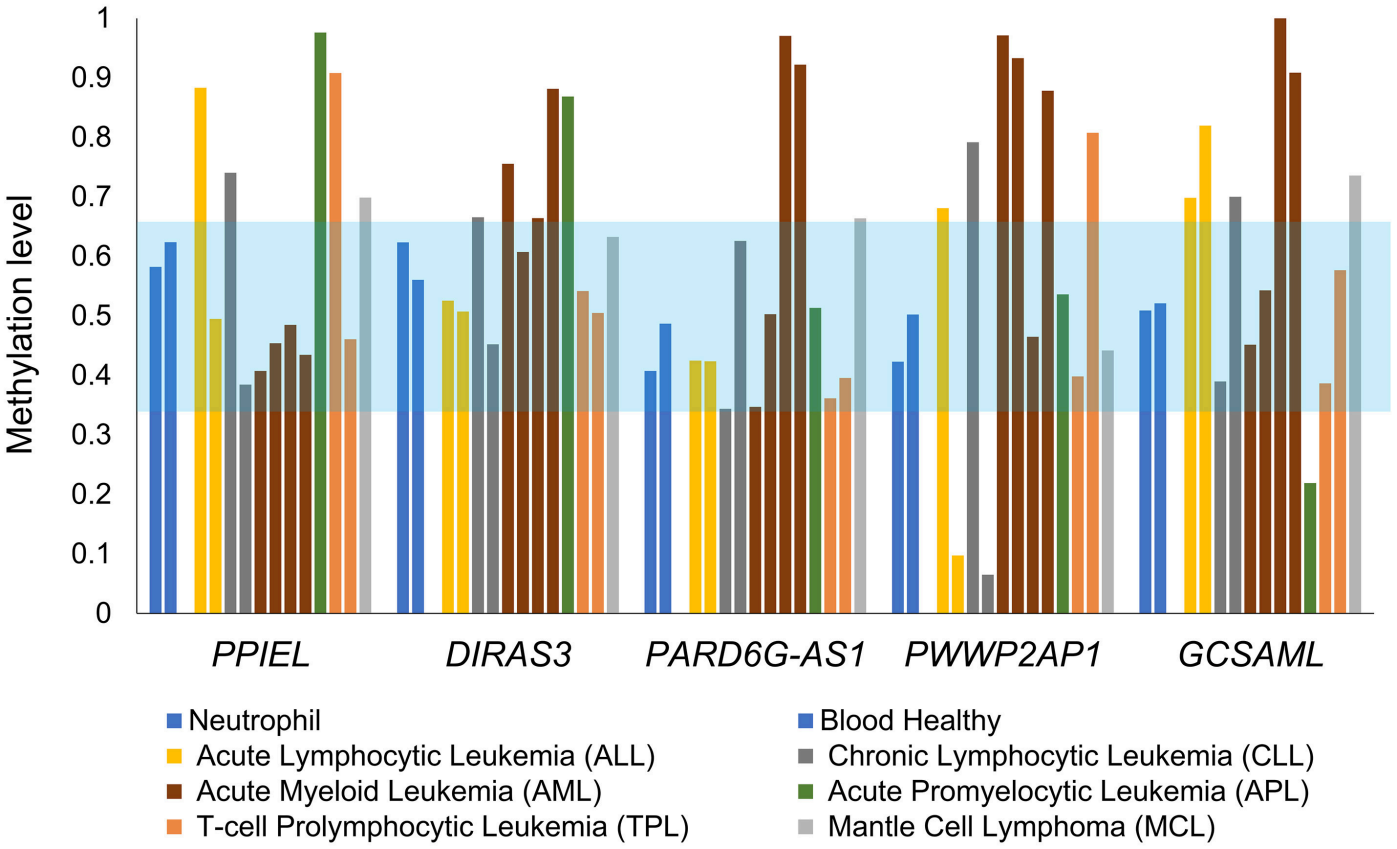
Aberrant 5^m^CpG DNA methylation at the new maternal iDMRs in hematopoietic cancers. The 5^m^CpG DNA methylation statuses at the *PARD6G-AS1* and *GCSAML* iDMRs are perturbed in methylomes of hematopoietic cancers, displaying either hypo- or hypermethylated profiles. Abnormal patterns are also observed at the known *PPIEL* and *DIRAS3* iDMRs, located on chromosome 1, which were used as a reference set. The light blue zone represents the expected intermediate methylation range (0.35–0.65). Each methylome is represented by a different color of bar.

The methylation statuses at the *PARD6G-AS1* and *GCSAML* iDMRs were perturbed in the methylomes of hematopoietic cancers, with both hypo- and hypermethylated profiles observed in different types of malignancies (Figure 9). Similar patterns of dysregulation were found in the known *PPIEL* and *DIRAS3* iDMRs located on the same or different chromosomes than the novel *PARD6G-AS1* and *GCSAML* iDMRs, respectively (Figure 9). Thus, the new *PARD6G-AS1* and *GCSAML* imprinted loci undergo cancer-associated epigenetic changes in hematopoietic malignancies.

### The 5^m^CpG epigenetic imprints are decoupled from parent-of-origin expression effects in multiple human tissues

Given that an iDMR can control the parent-of-origin-dependent monoallelic expression of a cluster of genes, we searched for evidence of ASE of genes located within 2.3 Mb of the *PARD6G-AS1* and *GCSAML* iDMRs in either direction. The 2.3 Mb range was selected because it matches the distance between the genes controlled by the *SNURF* iDMR (Eggermann et al., 2015). It is worth noting that certain known iDMRs are dissociated from POEs (Alves da Silva et al., 2016). We used two large sets of RNA-Seq experiments from 52 primary tissues to search for ASE across SNPs within 4.6 Mb chromosomal region spans, centered at the *PARD6G-AS1* and *GCSAML* iDMRs and the *PWWP2AP1* pseudogene. In the secondary GTEx set of RNA-Seq experiments, we designated a gene expression profile only if the evidence included at least three non-discordant SNPs with ≥12 reads each per tissue. Overall, we observed no signs of monoallelic expression for *PARD6G-AS1* or *GCSAML* and no transcriptional activity of the *PWWP2AP1* pseudogene in either the secondary (Dataset S2A) or the primary (Dataset S2C) source of RNA-Seq experiments. *PARD6G-AS1* was expressed biallelically in six tissues: brain amygdala (4 SNPs), brain putamen (5 SNPs), heart atrial appendage (6 SNPs), pituitary (4 SNPs), testis (3 SNPs) and thyroid (7 SNPs) (Dataset S2D: 4.6 Mb screen analysis). *GCSAML* was expressed biallelically in five tissues: visceral adipose tissue (omentum) (4 SNPs), brain - cerebellar hemisphere (6 SNPs), brain cerebellum (4 SNPs), prostate (6 SNPs), and testis (6 SNPs).

In humans, certain iDMRs can control the expression of genes that are up to 2.3 Mb away in either direction (Eggermann et al., 2015). Therefore, we searched the GTEx dataset for signs of monoallelic expression of genes neighboring the novel iDMRs (Dataset S2D). On chromosome 1, we found evidence of tissue-specific monoallelic expression of *ZNF124* (lung; 5 SNPs) and *OR2L13* (testis; 6 SNPs). *ZNF124* and *OR2L13* are located 363 kb upstream and 419 kb downstream, respectively, of the *GCSAML* iDMR (Dataset S2D). The *ZNF124* locus has no overlapping hemimethylated CGIs in normal tissues, including lung tissue (Figure S4A). In the placenta (Hamada et al., 2016), there is evidence of biallelic expression of *ZNF124* (Figure S4B, Dataset S2D).

On the other hand, the *OR2L13* locus has a predicted CGI-bearing AMR overlapping the promoter region (Figure S5A) in blood, spleen, lung, liver, esophagus, and brain methylomes, as well as two intergenic DMRs, predicted to be maternal in the placenta (Figure S5B). Nevertheless, the CGI-bearing AMR is hypomethylated in gamete, blastocyst, placenta, thymus, and spinal cord methylomes and hypermethylated in hESC-derived CD56^+^ ectoderm and mesoderm and in hESC-derived CD184^+^ endoderm cell cultures (Figure S5A). Unfortunately, there are no BS-Seq or 450 K methylomes available from testis tissue; thus, we cannot establish the methylation status in that tissue.

On chromosome 18, we found evidence of pituitary-specific expression of the *RP11-567M16.1-LOC284240* locus (8 SNPs) (Dataset S2D). The *RP11-567M16.1-LOC284240* locus encodes a lincRNA. At loci 18.6 and 4.9 kb upstream, we annotated two predicted paternal DMRs, neither of which exhibited a constitutively or a tissue-specific hemimethylated profile (Figure S6). Because no BS-Seq or 450 K methylomes are available for the pituitary, we cannot rule out the possibility of a pituitary-specific iDMR. Importantly, a CGI (*LOC284240* CGI-2) located 3.2 kb upstream from the *LOC284240* locus shows an intermediate methylation status with paternal, rather than maternal, methylation asymmetry in gametes (Figure S7) in BS-Seq methylomes from various tissues. The hemimethylated status at *LOC284240* CGI-2 is not constitutive because CGI-2 is hypermethylated in spleen, liver, thymus, and hESC-derived cells (Figure S7). Querying the primary RNA-Seq experiments from lung (*ZNF124*), testis (*OR2L13*) and pituitary (*LOC284240*) tissues yielded >10 reads only for the *ZNF124* SNPs, with no evidence of biallelic expression (Dataset S2E).

From the 4.6 Mb screen described above, we selected the SNPs with MAF ≥0.1 that exhibited ≥80 reads in the GTEx subset (*n* = 47; Dataset S2F: Heatmap of top SNPs) and queried them against the primary set of 2,164 RNA-Seq experiments. We confirmed the evidence of biallelic expression with ≥20 reads for 46 SNPs, including the four SNPs in *PARD6G-AS1* (Dataset S2G: ASE top biallelic SNPs). The only discordant profile found was for the SNP rs75287701 in the *HSBP1L1* gene, which showed monoallelic expression in 10 tissues. The discordance is apparently due to the low allele G frequency (0.144) in the general population. Lastly, we observed no evidence of RNA-Seq expression for the *PWWP2AP1* pseudogene in either the secondary or the primary subset of RNA-Seq experiments. Nevertheless, the neighboring loci are biallelically expressed in multiple tissues (Dataset S2D).

### Epigenomic chromatin segmentation at the *PARD6G-AS1* and *GCSAML* iDMRs

**Figure 10:**
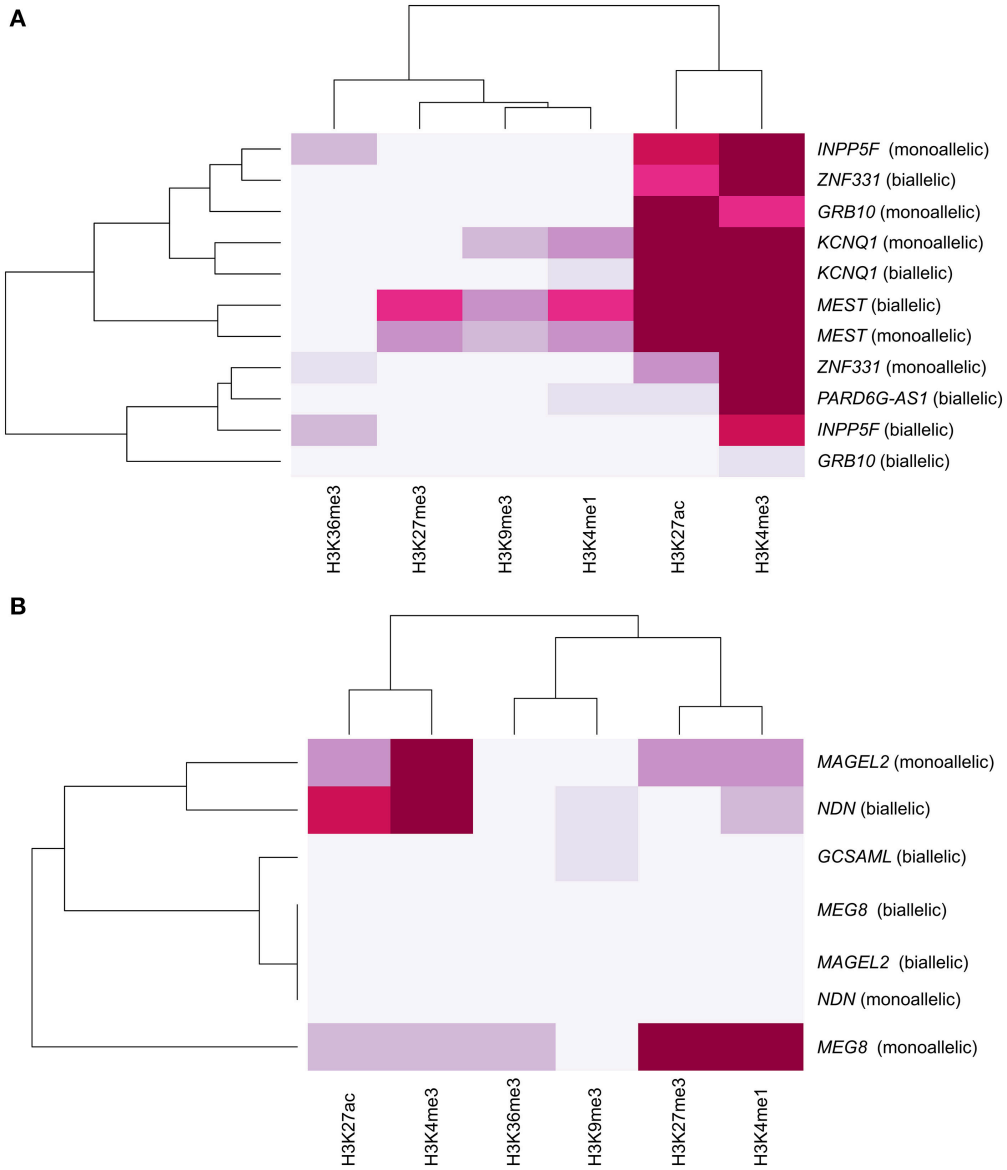
Absence of a tissue-specific histone modification enrichment signature at the *PARD6G-AS1* and *GCSAML* iDMRs. Hierarchical clustering analysis of the histone modification enrichment profiles associated with maternal **(A)** gametic and **(B)** secondary iDMRs. The color intensity is correlated with the magnitude of the histone modification enrichment observed in tissues in which the genes are expressed either monoallelically or biallelically. Differential enrichment signatures in tissues with monoallelic expression are revealed for the *GRB10, MEG8*, and *MAGEL2* iDMRs. The *PARD6G-AS1* and *GCSAML* iDMRs were associated with contrasting chromatin segmentation marked by enrichment of activation and repression marks, respectively.

**Figure 11:**
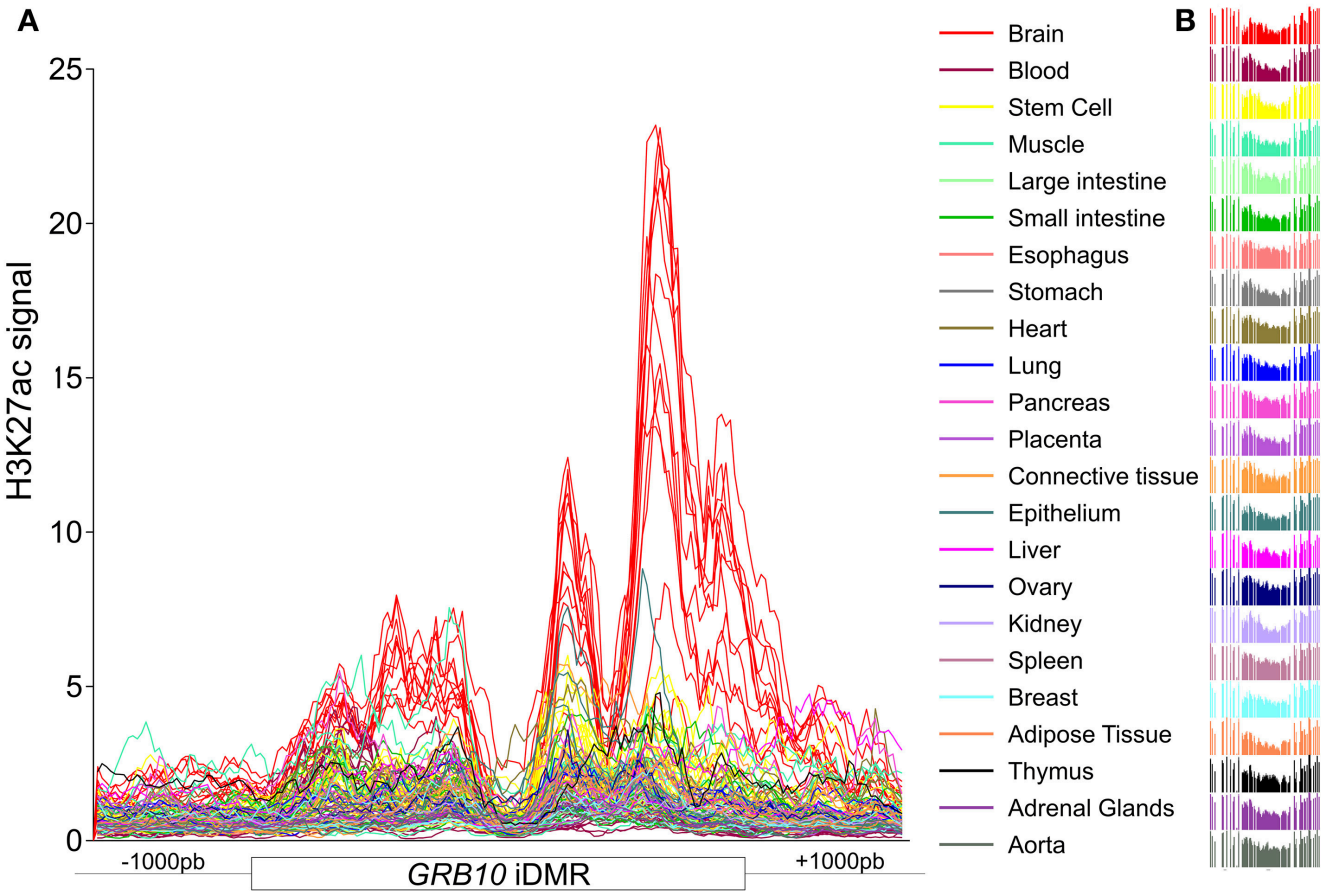
H3K27ac is differentially enriched at the *GRB10* maternal iDMR in the brain. **(A)** Distribution of the H3K27ac activation mark across the *GRB10* maternal iDMR in 22 tissues and one stem cell line. **(B)** A constitutively hemimethylated status is observed in all tissue methylomes. The line traces in **(A)** correspond in color to the tissue methylation profiles in **(B)**. Despite the constitutive hemimethylated status, there is a brain-specific enrichment of H3K27ac, an activation mark, at the *GRB10* iDMR; this enrichment is taken to be a differential signature of the active paternal allele in the brain.

Constitutive iDMRs (e.g., those that are stably maintained through mitosis in many tissues and cell lines) can regulate ASE of the underlying gene(s) in an isoform-specific and tissue-specific manner. For example, the oocyte-derived *GRB10* intronic iDMR (Arnaud et al., 2003), depicted in Figure S3, regulates the imprinting of the paternal and maternal *GRB10* alleles in a highly tissue- and isoform-specific manner (Blagitko et al., 2000; Yoshihashi et al., 2000; Mergenthaler et al., 2001)*. GRB10* is biallelically expressed in multiple tissues (Blagitko et al., 2000; Monk et al., 2009; Babak et al., 2015; Baran et al., 2015), including the growth plate cartilage (McCann et al., 2001), which is the tissue most important for linear growth. Importantly, there is alternate monoallelic expression in brain (transcribed from the paternally transmitted allele), skeletal muscle (from the maternal gamma 1 and gamma 2 isoform alleles) (Blagitko et al., 2000; Yoshihashi et al., 2000), human lymphocyte/rodent somatic hybrid cells (from the maternal allele) (Yoshihashi et al., 2000), and placental villous trophoblasts (from the maternal allele) (Monk et al., 2009). To gain insight into the epigenomic architecture regulating this class of POEs and to explore the possibility of the *PARD6G-AS1* and *GCSAML* iDMRs similarly controlling ASE, we compared the chromatin segmentation enrichment states achievable by these two constitutive maternal iDMRs through chemically modified histones. We used data from the integrative analysis of 111 reference epigenomes (Roadmap Epigenomics Consortium et al., 2015). We cross-referenced those data with the combinatorial histone signatures associated with five known maternal gametic iDMRs (Dataset S1G, Figure 10A) and three maternal secondary iDMRs (Figure 10B) in tissues where the corresponding genes are expressed either monoallelically or biallelically (Babak et al., 2015; Baran et al., 2015; Dataset S2B). We identified differential enrichment signatures for the iDMRs of *INPP5F, ZNF331, GRB10, KCNQ1, MAGEL2*, and *MEG8* in tissues with monoallelic expression (Figure 10). For the *PARD6G-AS1* and *GCSAML* iDMRs, genes for which monoallelic expression was absent from several tissues, we did not identify a tissue-specific enrichment signature (Figure 10). The *PARD6G-AS1* iDMR showed a chromatin segmentation enrichment with the H3K4me1, H3K4me3, and H3K27ac activation marks (Figure 10). The *GCSAML* iDMR showed enrichment of the H3K9me3 repressive mark. For the *GRB10* maternal gametic iDMR, we observed a brain-specific differential enrichment of the H3K27ac activation tag (Figure 11), which we propose to be associated with the differential reading of the 5^m^CpG imprints in the brain.

### iDMR genotype-phenotype associations

By cross-referencing SNPs in a broad range of phenotypes from large-scale GWAS repositories, we found the lead variant rs1050919 in the *PARD6G-AS1* iDMR to exert cis methylation QTL effects 113,318 bp away, on the *TXNL4A* gene at the chr18:77793182 hg19 coordinate cg04727522 probe, in frontal cortex and caudal pons (genome-wide *p*-value 3.39 × 10^−8^) (Dataset S3B: iDMR effect SNPs; and Dataset S3C: Effect and proxy SNP MAF). We note that H3K4me1, H3K4me3, and H3K27ac all contribute to the promoter-enhancer chromatin active state assignment at the lead variant rs1050919 in multiple tissues (Dataset S3D: rs1050919 chromatin states). We also found 38 proxies (linkage disequilibrium *r*^2^ ≥ 0.8) of two other lead SNPs (rs56357216 and rs1106086) in the *PARD6G-AS1* iDMR exerting the same cis-meQTL effect in temporal cortex (*p* < 1.76 × 10^−8^) (Datasets S3B,C). These two lead variants are associated with an active TSS regulatory chromatin state in >20 epigenomes (Dataset S3E: rs56357216 chromatin states and Dataset S3F: rs1106086 chromatin states). On the other hand, the rs7550918 proxy of the lead variant rs10925069 at the *GCSAML* iDMR showed a negative association with platelet counts (*p* = 3.00 × 10^−11^) (Dataset S3B). H3K4me1, H3K4me3, H3K27ac, and H3K9ac contribute to the chromatin active state assignment at the lead SNP rs10925069 in blood cells and only a few other tissues (Dataset S3G: rs10925069 chromatin states).

## Discussion

We demonstrated the occurrence of maternally inherited 5^m^CpG imprints at the DMRs linked to the non-coding RNA *PARD6G-AS1* and the protein-coding *GCSAML* gene. The 5^m^CpG imprints are oocyte-derived (*PARD6G-AS1* iDMR) or somatic (*GCSAML* iDMR), and their inheritance is dissociated from POEs in at least 10 adult somatic tissues, as determined by RNA-Seq profiles that were robustly consistent with biallelic expression across multiple informative SNPs. In the multitissue transcriptomes investigated, we did not see evidence for monoallelic expression from the parental alleles at SNPs that are isoform-specific or those that overlap isoforms or are in overlapping genes. Our study has a critical limitation regarding the number of informative SNPs covering the entire set of known or predicted genes included in each of three 4.6 Mb genomic regions that were screened for signs of allele expression profiles. The coverage rate of genes with informative SNPs ranged from 30% (*PWWAP2AP1*) to 59% (*PARD6G-AS1* and *GCSAML*) (Dataset S2H: Effective gene coverage). The limitation derives from the conservative and stringent criteria used to call the allele transcriptional profiles of genes. Specifically, we reported only unambiguous evidence supported by at least three informative SNPs per gene per body tissue. The findings do not rule out the possibility that, in other datasets with considerably higher read depths, the encompassed genes that our analysis found uninformative may exhibit profiles consistent with monoallelic expression. Moreover, our approach cannot rule out the possibility of monoallelic expression in tissues in which the levels of expression are below the limit of detection of 12 reads per SNP accepted in our experimental design, or in tissues that are not covered by the atlas set of 2,164 SRA RNA-Seq experiments. Similarly, we cannot exclude the possibility that the iDMRs are associated with the expression from the paternal allele of coding and non-coding genes that are over 2.3 Mb away in either direction, which is the greatest physical distance between any known iDMR and the underlying controlled gene(s) (e.g., the *SNRPN*/*SNURF* iDMR) (Eggermann et al., 2015).

A second significant limitation of the present study is that the experimental setting does not permit us to assess cell-specific differences in 5^m^CpG methylation levels, RNA-Seq allele expression, or enrichment of histone modifications within the same homogeneous cells, which may add considerable noise to the tissue-specificity analysis reported here. We recognize that the majority of experiments on 5^m^CpG DNA methylation, RNA-Seq and ChIP histone modifications from public repositories are conducted with tissue samples (e.g., a heterogeneous mixture of cell types), which may confound our findings. It is possible, for example, that a given gene is monoallelically expressed in a parent-of-origin-dependent manner in just one cell type (e.g., platelets, lymphocytes or monocytes in peripheral blood) while the expression profile in whole blood is consistently biallelic. Moreover, we are aware that metabolic differences between cultured tissues and tissues collected by biopsy from living or necropsy donors (e.g., the GTEx study) must be kept in mind when trying to replicate our results with other biosamples.

The maternal origin of the 5^m^CpG imprints at the *PARD6G-AS1* gametic iDMR enables a direct interrogation of the parental origin of the nondisjunction in trisomy 18 index cases without the need to test gDNA from the progenitors, since the methylation levels at the iDMR will be differential in cases of paternally (skewed hypomethylation; ≤0.33) versus maternally inherited (skewed hypermethylation; ≥0.66) supernumerary chromosome 18. This parent-of-origin-dependent epigenetic feature of iDMRs was recently explored in the identification of the parental origin of trisomy 21 nondisjunction on the basis of the *WRB* gametic iDMR (Alves da Silva et al., 2016).

We note that the maternally derived 5^m^CpG imprints at the *PARD6G-AS1* and *GCSAML* iDMRs displayed concordant methylation levels in 90 human placentas, with no evidence for epipolymorphism in 5^m^CpG DNA methylation level. The finding is consistent with the rapidly evolving concept that only placenta-specific maternal iDMRs represent stochastic (e.g., switchable) epipolymorphic traits (Yuen et al., 2009; Hamada et al., 2016; Hanna et al., 2016; Sanchez-Delgado et al., 2016a). The epipolymorphism in placenta-specific maternal iDMRs is interindividual (e.g., across individual placentas), and it is not associated with distinct profiles generated by sampling different sections of the same placenta (Yuen et al., 2009).

Regarding other possible biological relevance of the 5^m^CpG imprints, we note that significant abnormal hypomethylation has been reported for individual CpG sites located within the *PARD6G-AS1* gametic iDMR in some patients with one of three imprinted defects. Between 3 and 9 CpG sites are affected in Beckwith-Wiedemann syndrome (the lowest *p* = 1.01 × 10^−19^) and transient neonatal diabetes (4.38 × 10^−71^) patients, respectively, compared with matched control subjects (Docherty et al., 2014), while two encompassing CpG sites are impacted in pseudohypoparathyroidism patients with *GNAS* cluster imprinting defects (Rochtus et al., 2016).

An epigenome-wide association study of aggressive behavior showed a significant positive relationship (*p* = 9.0 × 10^−7^, FDR = 0.18) between the increase in methylation (over the average intermediate level; mean 0.35, *SD* = 0.096) in venous blood at the cg06092953 site (located 135 bp downstream from the newly validated *PARD6G-AS1* maternal iDMR) and the increase in residual aggression scores across the entire Netherlands Twin Register (van Dongen et al., 2015). Thus, we hypothesize that heritability influences the aforementioned epigenetic association with aggression.

Similarly, the methylation status at the cg18973878 site (located 179 bp upstream from the non-coding *PARD6G-AS1* maternal iDMR) was recently identified as a cis-meQTL, significantly affected when the rs11659843 SNP minor allele T is transmitted from the father (genome-wide *p*-values of 1.95 × 10^−7^−5.32 × 10^−21^) (Cuellar Partida et al., 2017). The SNP exerting the cis-meQTL effect is intronic to the protein-coding *PARD6G* gene, and it maps 79,598 bp (hg19) away from the *PARD6G-AS1* maternal iDMR, which evidently does not overlap the *PARD6G* gene.

We also note that within the *PARD6G-AS1* maternal iDMR, evidence for haplotype-dependent allele-specific 5^m^CpG sites has been reported in several methylomes (Do et al., 2016). We propose that the epi-haplotypes above occur in a parent-of-origin-dependent manner and, thus, that they would be useful in studying susceptibility to complex imprinted genetic diseases (Sanchez-Delgado et al., 2016b).

We notice that a significant change toward a hypermethylated status at four CpG sites (β differences ranged from +0.15 to +0.22) within the chromosomal region corresponding to the *GCSAML* secondary maternal iDMR has been reported in postmortem prefrontal cortex in one case of Klinefelter syndrome (47,XXY karyotype) versus control males (Viana et al., 2014), and the perturbation was subsequently confirmed in a second study using venous blood samples from five patients with Klinefelter syndrome (Wan et al., 2015). The perturbed 5^m^CpG sites are cg05639522, cg18198730, cg11166453, and cg07313626 (genome-wide *p*-values ranged from 3.85 × 10^−5^−5.07 × 10^−7^). The first Klinefelter case had comorbid schizophrenia along with a notably reduced cerebellum mass, and the β difference in methylation levels between the index case and sex-matched control samples was 0.16 (*p* = 1.35 × 10^−6^) (Viana et al., 2014). In both studies, the difference from female controls was nonsignificant. Thus, the supernumerary X chromosome in the index patients may be the direct cause of the disturbance in the imprinted intermediate methylation at this autosomal locus. It follows that the epigenetic perturbance may be linked to the development and evolution of psychotic spectrum conditions, as has been suggested for differential methylation of the candidate imprinted *GNAL* gene with possible maternal effects in the linkage to psychosis (reviewed in Crespi, 2008). On the other hand, we showed herein that the supernumerary autosomes 13, 18, and 21 cause significant changes in the asymmetrical methylation statuses at this iDMR in the placenta.

We hypothesize that the *GCSAML* maternal iDMR regulates the lung-specific, monoallelic expression of *ZNF124* from the paternal allele. Addressing this possibility will require establishing the parental origin of the expressed allele(s) and carrying out functional genetics studies to determine the effects of, for example, permanently perturbing the distant *GCSAML* iDMR. *ZNF124* encodes a potential transcriptional factor, and no variants have been flagged in GWAS. *ZNF124* is a candidate gene for the Dandy-Walker complex at 1q44 (Poot et al., 2007). Interestingly, the reported Dandy-Walker complex propositus had a 5 Mb segmental aneuploidy (spanning the *ZNF124, GCSAML*, and *OR2L13* genes) of the 1q44 → qter region due to a paternal t(1;20) (q44;q13.33). Thus, in the scenario of maternal genomic imprinting, the monoallelic expression of the predicted paternal allele will be precluded in the haploinsufficient patient, who would manifest the presumed imprinting defect as a pathogenic null mutation.

We view the testis-specific monoallelic expression of *OR2L13* as indicative of allele exclusion rather than genomic imprinting coupled to the distant *GCSAML* iDMR. The reasons are as follows: the *OR2L13* gene encodes an olfactory receptor, and the monoallelic expression of olfactory receptors is mediated by epigenetic mechanisms leading to allelic exclusion (Monahan and Lomvardas, 2015). It is worth noting that the abovementioned 5 Mb segmental aneuploidy of the 1q44 → qter region also encompasses the cluster of olfactory receptor genes that includes *OR2L13* (Poot et al., 2007).

To date, the placenta is the only confirmed source of tissue-specific iDMRs (Hanna et al., 2016). The candidate *LOC284240* paternal iDMR occurs in the skin, lung, fetal brain cortex, fetal spinal cord, and placenta; therefore, it exemplifies a new variable type of iDMR. We propose that the observed pituitary-specific monoallelic expression of the *RP11-567M16.1-LOC284240* locus is coupled to the novel candidate paternal iDMR represented by *LOC284240* CGI-2, rather than to the distant *PARD6G-AS1* maternal iDMR.

Finally, we view as unusual the finding that the maternal secondary *GCSAML* iDMR is not associated with a primary iDMR within a 4.6 Mb genomic span. Canonical secondary iDMRs are found most frequently near functional gametic iDMRs. For example, in the case of the gametic IG-DMR and the secondary *MEG3*-DMR (15.9 kb apart), there is evidence of hierarchical regulation of imprinting in 14q32. The IG-DMR regulates the methylation state of the *MEG3*-DMR, but not vice versa (Beygo et al., 2015). Although our analysis does not rule out the possibility of the occurrence of a primary iDMR beyond the 2.3 Mb limits in either direction, it appears that the *GCSAML* iDMR is the second example in humans of a secondary iDMR that lacks a primary iDMR. The first reported iDMR lacking a known constitutive, primary iDMR was the maternal *ZDBF2* iDMR (Kobayashi et al., 2013). In contrast to the *GCSAML* iDMR, the orphan *ZDBF2* iDMR regulates the monoallelic expression of the *ZDBF2* gene in at least 46 tissues (Dataset S2B; Baran et al., 2015).

## Concluding remarks

We identified 125 constitutively hemimethylated domains in the human genome. Twenty-nine domains displayed asymmetrical 5^m^CpG imprints in gametes, including 18 oocyte-derived and 11 spermatozoon-derived epigenetic marks, and those 29 domains were classified as candidate gametic (primary) iDMRs. The remaining 96 hemimethylated domains were classified as candidate secondary iDMRs. We established the maternal inheritance of the 5^m^CpG constitutive imprints at the *PARD6G-AS1* and *GCSAML* iDMRs and their disassociation from POEs in the allelic transcriptional expression profiles across a 4.6 Mb domain around each iDMR in multiple human primary tissues. Our results also revealed the occurrence of a constitutively hemimethylated, nonimprinted domain at *PWWP2AP1* CGI-1 with oocyte-derived methylation asymmetry. The maternally inherited 5^m^CpG imprints at the *PARD6G-AS1* and *GCSAML* iDMRs are perturbed in hematopoietic cancers.

## Web resources

The URLs for data presented herein are as follows:

UCSC Genome Browser, https://genome.ucsc.edu/

BLUEPRINT project, http://www.blueprint-epigenome.eu/

GTEx Portal, https://www.gtexportal.org/

NCBI SRA, https://www.ncbi.nlm.nih.gov/sra/

NCBI GEO, http://www.ncbi.nlm.nih.gov/geo/

Roadmap Epigenomics Browser, http://www.roadmapepigenomics.org/

WashU Epigenome Browser, http://epigenomegateway.wustl.edu/

FANTOM ZENBU Browser, http://fantom.gsc.riken.jp/zenbu/

dbGaP, http://www.ncbi.nlm.nih.gov/gap

PhenoScanner. http://www.phenoscanner.medschl.cam.ac.uk/phenoscanner

e-GRASP, http://www.mypeg.info/egrasp

HaploReg, http://compbio.mit.edu/HaploReg

PheGen*I*; https://www.ncbi.nlm.nih.gov/gap/phegeni

Geneimprint, http://www.geneimprint.com/

OMIM, http://www.omim.org

R software package, http://www.R-project.org

## Ethics statement

This study complied with the recommendations of the Ethics Committee of the Faculdade de Medicina de Campos, Brazil (approval code FR-278769). The goal of the study was to identify imprinted differentially methylated regions for the development of molecular genetic tests. The study did not involve physical or history examinations of subjects or laboratory testing for a genetic disease, as the subjects' DNA was used exclusively for DNA marker validation purposes. Healthy volunteer subjects were sampled by convenience or accessibility. Peripheral blood samples from participating adult subjects were collected with the written informed consent in compliance with the Declaration of Helsinki. For the youth, written consent was given by the legally authorized next of kin on behalf of the participants.

## Author contributions

EM-A, FBM, GdSMA, RdSFJ, PTMR, CdSF, DTM, JTdS: conceived and designed experiments. GdSMA, RdSFJ, PTMR, FMB, CCFA, AFAdS, CdSF, EM-A: performed comparative screening of candidate iDMR in methylomes. GdSMA and TLdS: performed MSRE-PCR and SNuPE genotyping. RdSFJ, CdSF, VR, EM-A: developed R and Excel scripts. RdSFJ, CdSF, EM-A: carried out the GTEx analysis. DTM, ATdS, CFOdF, ABG: performed SRA RNA-Seq analysis. PTMR: executed the placenta methylome analysis. JTdS, RdSFJ, CFOdF, EM-A: searched chromatin segmentation enrichment signatures. DTM, CdSF, PTMR, RdSFJ, JTdS, CFOdF, TLdS, EM-A: prepared figures. EM-A: carried out comprehensive computational analyses; contributed biological samples, reagents, and materials; and wrote the manuscript.

### Conflict of interest statement

The authors declare that the research was conducted in the absence of any commercial or financial relationships that could be construed as a potential conflict of interest.

## Supplementary material

Dataset S1 Information about the novel candidate imprinted DMRs. Seven spreadsheets: **(A)** List of the 39 BS-Seq methylomes used to extract the AMR records; **(B)** List of the new candidate iDMR reported in this study; **(C)** List of known constitutive imprinted DMRs (Okae et al., 2014) used to set the investigative inclusion criterion of ≥16 overlapping AMR records in each candidate iDMR; **(D)** Information about the primers, MSRE-PCR triplex assays, and SNPs used to determine the parent-of-origin of the 5^m^CpG imprints in the *PARD6G-AS1, PWWP2AP1*, and *GCSAML* candidate iDMRs; **(E)** List of 90 GEO entries for 450 K methylation arrays from healthy; preeclampsia; and trisomy 13 (Patau syndrome), 18 (Edwards syndrome) and 21 (Down syndrome) placentas and list of BS-Seq methylomes from healthy (*n* = 3) placentas; **(F)** List of BS-Seq methylomes from hematopoietic cancers from the BLUEPRINT project (Adams et al., 2012); **(G)** Roadmap Epigenomics samples; **(H)** Reference set of iDMRs used in the hierarchical clustering analysis of histone modification enrichment.

Dataset S2 Overall evidence of allele-specific expression (ASE) from RNA-Seq experiments. Six spreadsheets: **(A)** Information about the 8,555 RNA-Seq experiments in 51 tissues from the GTEx project (release V7; *n* = 9,931 RNA-Seq entries extracted with the dbGaP run selector tool), used as a secondary source; **(B)** Sorting and classification of 40 known imprinted genes (Babak et al., 2015; Baran et al., 2015) by the patterns of ASE across informative SNPs using the GTEx data and classification criteria described in the present study; **(C)** Information about the 2,164 RNA-Seq experiments in 25 tissues used as the primary source. Jointly, the sets of RNA-Seq experiments listed in **(A,C)** represent an atlas of 52 tissues; **(D)** Sorting and classification of the genes located up to 2.3Mb in either direction from the *PARD6G-AS1, PWWP2AP1*, and *GCSAML* genes by the patterns of ASE across informative SNPs using the GTEx data; **(E)** Allele-specific expression of *ZNF124* yielding at least 10 reads per SNP in primary RNA-Seq experiments from lung tissue; **(F)** List of the top lead SNPs (MAF > 0.1) selected from the results in **(D)** and heatmap of the distribution of the informative RNA-Seq experiments yielding at least 20 reads per SNP; **(G)** Evidence of biallelic expression across the lead SNPs listed in **(F)**; **(H)** Effective gene coverage according to the GTEx expression analysis.

Dataset S3 PhenoScanner, e-GRASP, PheGen*I*, and HaploReg lookup of genotype-phenotype genome-wide associations for SNPs in the *PARD6G-AS1* and *GCSAML* iDMRs. Seven spreadsheets: **(A)** Complete set of SNPs in each iDMR; **(B)** The list of SNPs and proxies found in association with traits at genome-wide significance; **(C)** Gene positions, minor alleles and global MAFs for the SNPs and proxies with genome-wide significance in GWAS; regulatory chromatin states at the lead variants rs1050919 **(D)**, rs56357216 **(E)**, rs1106086 **(F)**, and rs10925069 **(G)**.

Figure S1 Constitutive unmethylated status at the *PPP2R3B* predicted promoter region. X- chromosome ideogram; physical positions and domain features of the *PPP2R3B* locus depicting the methylation status at CpG sites across a 40 kb long-span view (hg19; 329240–369239). The methylation levels are represented on a scale from 0 to 1 (hypomethylated to hypermethylated). The image is centered on CGI-6, localized in the *PPP2R3B* predicted promoter region. The light green ticks represent the positions of the CpG sites. The *PPP2R3B* gene is transcribed from the minus DNA strand. The annotated features are (from top to bottom) the segmental duplication in chrY (chrY:231384 track), the exon-intron organization of the principal isoform, the evidence of small non-coding RNAs (sncRNAs) (DASHR RNA [neg] track), the FANTOM5 transcriptional start sites (TSS peaks track) (Lizio et al., 2017), the Ensembl Regulatory Build predicted promoters (Ensembl Reg Build track) (Cunningham et al., 2015), CpG islands and CGI-bearing AMRs (this study) and the comparative custom Fang_AMR track (Fang et al., 2012). A constitutive unmethylated status is observed across *PPP2R3B* CGI-6 in gametes, as well as in healthy (golden ticks) and cancerous (orange ticks) adult tissues. The chromosomal position of the region corresponding to the *Hpa*II-sensitive amplimer (PCR results track) is highlighted in light blue within *PPP2R3B* CGI-6.

Figure S2 Validation of the intermediate methylation statuses at the *PARD6G-AS1, PWWP2AP1*, and *GCSAML* DMRs by MSRE-PCR triplex assays. Representative methylation profiles before and after digestion with the methylation-sensitive *Hpa*II restriction enzyme. **(A)** *PARD6G-AS1* DMR, **(B)** *PWWP2AP1* DMR, and **(C)** *GCSAML* DMR. Each profile generated from uncut DNA consists of two control products (left and right peaks) and a test product (peak in the middle). The control products correspond to the amplimers of a chromosomal region known to be 100% unmethylated in the human genome (left peak) (see Materials and Methods for details) and a chromosomal region bearing no *Hpa*II sites (right peak). The *PARD6G-AS1* DMR amplimer comprises the common indel variant rs11281142 (MAF > 0.4), and thus, a heterozygous sample yields two peaks representing the two variant alleles, one with and the other without the CTGTGGTGC insertion. The *GCSAML* DMR amplimer comprises two peaks (255 and 257 bp), consistent with the addition of a 3′-end A residue overhang to the PCR product, since the two-peak product occurs in 20 genomic DNA samples tested, and the two annotated, encompassing, one-base-pair indel variants rs533592199 (-/G), and rs773445240 (-/T) reported in dbSNP150 have not been detected in over 841,883 public exomes (NHLBI GO Exome Sequencing Project, 2012; Sulem et al., 2015; Chen et al., 2016; Lek et al., 2016; Narasimhan et al., 2016). Each panel corresponds to a different individual from one out of three representative nuclear families that were informative for at least one SNP. The biallelic methylation profile at the *PWWP2AP1* hemimethylated CGI DMR was confirmed using a second MSRE (*Hha*I) in two informative individuals (data not shown).

Figure S3 Constitutive hemimethylation statuses across the known *GRB10* maternal iDMR. **(A)** Chromosome 7 ideogram; physical positions and domain features of the *GRB10* locus showing the methylation status at CpG sites (golden ticks) across a 40 kb long-span view (hg19; chr7:50832014-50872013). The image is centered on the CGI localized in the *GRB10* predicted promoter region, labeled “*GRB10* maternal iDMR” (*GRB10* CGI-1), which maps to a predicted secondary promoter region upstream of the transcriptional start site of the short *GRB10* isoform. The *GRB10* locus is transcribed from the minus DNA strand. The light green ticks represent the positions of the CpG sites. The methylation levels are represented on a scale from 0 to 1 (hypomethylated to hypermethylated). The methylation levels across the CpG sites within the *GRB10* maternal iDMR range from 0.35 to 0.65 in adult somatic tissues. In the same region, however, there is asymmetrical methylation in gametes (hypermethylation in oocytes and hypomethylation in spermatozoa). **(B)** Cross-reference overlaps of the *GRB10* maternal iDMR with differentially methylated regions reported in control-disease methylomes and healthy placentas. The annotated features are (from top to bottom) the exon-intron organization of the principal and alternative isoforms, the evidence of large intergenic non-coding RNAs (lincRNA) and small non-coding RNAs (sncRNAs) (DASHR RNA [neg] track), the FANTOM5 transcriptional start sites (TSS peaks track) (Lizio et al., 2017), the Ensembl Regulatory Build predicted promoters (Ensembl Reg Build track) and transcriptional factor binding sites (TFBS summary track) (Cunningham et al., 2015), CpG islands and the comparative custom Fang_AMR track (Fang et al., 2012). The *GRB10* maternal iDMR overlaps the domain known be differentially methylated in a maternal-origin-dependent manner in blastocysts and the placenta (Okae et al., 2014; Hamada et al., 2016; Sanchez-Delgado et al., 2016a) (Okae_DMRs, Hamada_DMRs, Sanchez_DMRs_set:I and Sanchez_DMRs_set:II tracks, respectively). *GRB10* CGI-1 also intersects the domains reported to be hypomethylated in pseudohypoparathyroidism patients with *GNAS* cluster imprinting defects (Rochtus et al., 2016) (Rochtus_DMR track) and the maternally derived 5^m^CpG sites in placenta from (Yuen et al., 2011) (Yuen_5mCpG sites track). In the brain, the short *GRB10* isoform is expressed from the paternal allele from the promoter that overlaps the *GRB10* maternal iDMR. In the placenta, the expression of the long *GRB10* isoform is biased (0.79) toward the maternal allele (Hamada_biased_maternal_PL track) (Hamada et al., 2016), and it starts from the promoter region that overlaps *GRB10* CGI-2, which is constitutively hypomethylated.

Figure S4 The *ZNF124* locus has no overlapping hemimethylated CGIs. **(A)** Chromosome 1 ideogram; physical positions and domain features of the *ZNF124* locus showing the methylation status at CpG sites (golden ticks) across a 40 kb long-span view (hg19; chr1:247315500–247355499). The image is centered on the CGI located in the *ZNF124* predicted promoter region. The *ZNF124* locus is transcribed from the minus DNA strand. The light green ticks represent the positions of the CpG sites. The *ZNF124* CGI is hypomethylated in all shown BS-Seq methylomes. **(B)** Cross-reference overlaps of the predicted differentially methylated regions reported in healthy placentas. The annotated features are as in Figure S3. In the placenta, there is evidence of biallelic expression (Hamada et al., 2016).

Figure S5 The *OR2L13* locus has no overlapping hemimethylated CGIs. **(A)** Chromosome 1 ideogram; physical positions and domain features of the *OR2L13* locus showing the methylation status at the CpG sites (golden ticks) across a 40 kb long-span view (hg19; chr1:248098501–248138500). The light green ticks represent the positions of the CpG sites. The *OR2L13* CGI, localized in the *OR2L13* predicted promoter region, is hypomethylated in all shown BS-Seq methylomes analyzed (average level of 0.24). **(B)** Cross-reference overlaps of the predicted DMR reported in healthy placentas across a 70kb long-span view (hg19; chr1:248098401–248168400). In the placenta (Hamada-DMR track), there are two intergenic predicted maternal DMRs 56.3 and 65.6 kb downstream, respectively, of the *OR2L13* CGI. The annotated features are as in Figure S3.

Figure S6 The *RP11-567M16.1-LOC284240* locus exhibits a hemimethylated CGI. **(A)** Chromosome 18 ideogram; physical positions and domain features of the *RP11-567M16.1-LOC284240* locus showing the methylation status at the CpG sites (golden ticks) across a 40 kb long-span view (hg19; chr18:77317230–77357229). The image is centered at the *RP11-567M16.1-LOC284240* CGI-2 predicted promoter region. The light green ticks represent the position of the CpG sites. *RP11-567M16.1-LOC284240* CGI-2 is hemimethylated (average level of 0.53) in several BS-Seq methylomes, including those of the epidermis, esophagus, lung, fetal brain cortex, fetal spinal cord, and placenta tissues, with paternal rather than maternal methylation asymmetry in gametes (this study). The occurrence of a paternal primary candidate iDMR is consistent with the observation of monoallelic expression of the locus in the pituitary (this study). **(B)** Cross-reference for two predicted paternal DMRs (Hamada_DMRs, hamada_1st_trim, Sanchez_pDMR_PL, and Sanchez_pDMR_embryo tracks), none of which exhibit either a constitutive or a tissue-specific hemimethylation profile.

Figure S7 Predicted paternally-derived intermediate methylation at *LOC284240* CGI-2. Methylation levels across CGI-2 at the predicted promoter-flanking region of the *LOC284240* locus. Note the paternally-derived 5^m^CpG asymmetry in gametes and the intermediate methylation levels in BS-Seq methylomes from epidermis, lung, esophagus, gastric muscle, brain cortex, fetal spinal cord, and placenta. The hemimethylated status is not constitutive, since, in spleen, liver, blood, thymus, and the hESC-derived endodermal, mesodermal and endodermal cell lines, there is a bias toward hypermethylation.

## References

[B1] Adams D. Altucci L. Antonarakis S. E. Ballesteros J. Beck S. Bird A.. (2012). BLUEPRINT to decode the epigenetic signature written in blood. Nat. Biotechnol. 30, 224–226. 10.1038/nbt.2153 22398613

[B2] Alves da Silva A. F. Machado F. B. Pavarino E. C. Biselli-Perico J. M. Zampieri B. L. Da Silva Francisco Junior R.. (2016). Trisomy 21 Alters DNA methylation in parent-of-origin-dependent and -independent manners. PLoS ONE 11:e0154108. 10.1371/journal.pone.0154108 27100087 PMC4839675

[B3] Arnaud P. Monk D. Hitchins M. Gordon E. Dean W. Beechey C. V.. (2003). Conserved methylation imprints in the human and mouse GRB10 genes with divergent allelic expression suggests differential reading of the same mark. Hum. Mol. Genet. 12, 1005–1019. 10.1093/hmg/ddg110 12700169

[B4] Babak T. Deveale B. Tsang E. K. Zhou Y. Li X. Smith K. S.. (2015). Genetic conflict reflected in tissue-specific maps of genomic imprinting in human and mouse. Nat. Genet. 47, 544–549. 10.1038/ng.3274 25848752 PMC4414907

[B5] Baran Y. Subramaniam M. Biton A. Tukiainen T. Tsang E. K. Rivas M. A.. (2015). The landscape of genomic imprinting across diverse adult human tissues. Genome Res. 25, 927–936. 10.1101/gr.192278.115 25953952 PMC4484390

[B6] Barlow D. P. Bartolomei M. S. (2014). Genomic imprinting in mammals. Cold Spring Harb. Perspect. Biol. 6, 1–19. 10.1101/cshperspect.a018382 24492710 PMC3941233

[B7] Barrow T. M. Barault L. Ellsworth R. E. Harris H. R. Binder A. M. Valente A. L.. (2015). Aberrant methylation of imprinted genes is associated with negative hormone receptor status in invasive breast cancer. Int. J. Cancer 137, 537–547. 10.1002/ijc.29419 25560175 PMC4437845

[B8] Bernstein B. E. Stamatoyannopoulos J. A. Costello J. F. Ren B. Milosavljevic A. Meissner A.. (2010). The NIH roadmap epigenomics mapping consortium. Nat. Biotechnol. 28, 1045–1048. 10.1038/nbt1010-1045 20944595 PMC3607281

[B9] Beygo J. Elbracht M. De Groot K. Begemann M. Kanber D. Platzer K.. (2015). Novel deletions affecting the MEG3-DMR provide further evidence for a hierarchical regulation of imprinting in 14q32. Eur. J. Hum. Genet. 23, 180–188. 10.1038/ejhg.2014.72 24801763 PMC4297900

[B10] Blagitko N. Mergenthaler S. Schulz U. Wollmann H. A. Craigen W. Eggermann T.. (2000). Human GRB10 is imprinted and expressed from the paternal and maternal allele in a highly tissue- and isoform-specific fashion. Hum. Mol. Genet. 9, 1587–1595. 10.1093/hmg/9.11.1587 10861285

[B11] Castel S. E. Levy-Moonshine A. Mohammadi P. Banks E. Lappalainen T. (2015). Tools and best practices for data processing in allelic expression analysis. Genome Biol. 16:195. 10.1186/s13059-015-0762-6 26381377 PMC4574606

[B12] Chen R. Shi L. Hakenberg J. Naughton B. Sklar P. Zhang J.. (2016). Analysis of 589,306 genomes identifies individuals resilient to severe Mendelian childhood diseases. Nat. Biotechnol. 34, 531–538. 10.1038/nbt.3514 27065010

[B13] Chen Y. A. Lemire M. Choufani S. Butcher D. T. Grafodatskaya D. Zanke B. W.. (2013). Discovery of cross-reactive probes and polymorphic CpGs in the Illumina Infinium HumanMethylation450 microarray. Epigenetics 8, 203–209. 10.4161/epi.23470 23314698 PMC3592906

[B14] Chess A. (2016). Monoallelic Gene Expression in Mammals. Annu. Rev. Genet. 50, 317–327. 10.1146/annurev-genet-120215-035120 27893959

[B15] Choufani S. Shapiro J. S. Susiarjo M. Butcher D. T. Grafodatskaya D. Lou Y.. (2011). A novel approach identifies new differentially methylated regions (DMRs) associated with imprinted genes. Genome Res. 21, 465–476. 10.1101/gr.111922.110 21324877 PMC3044860

[B16] Cooper W. N. Constancia M. (2010). How genome-wide approaches can be used to unravel the remaining secrets of the imprintome. Brief. Funct. Genomics 9, 315–328. 10.1093/bfgp/elq018 20675687

[B17] Court F. Martin-Trujillo A. Romanelli V. Garin I. Iglesias-Platas I. Salafsky I.. (2013). Genome-wide allelic methylation analysis reveals disease-specific susceptibility to multiple methylation defects in imprinting syndromes. Hum. Mutat. 34, 595–602. 10.1002/humu.22276 23335487

[B18] Court F. Tayama C. Romanelli V. Martin-Trujillo A. Iglesias-Platas I. Okamura K.. (2014). Genome-wide parent-of-origin DNA methylation analysis reveals the intricacies of human imprinting and suggests a germline methylation-independent mechanism of establishment. Genome Res. 24, 554–569. 10.1101/gr.164913.113 24402520 PMC3975056

[B19] Crespi B. (2008). Genomic imprinting in the development and evolution of psychotic spectrum conditions. Biol. Rev. Camb. Philos. Soc. 83, 441–493. 10.1111/j.1469-185X.2008.00050.x 18783362

[B20] Cuellar Partida G. Laurin C. Ring S. Gaunt T. R. Relton C. L. Davey Smith G. (2017). Imprinted loci may be more widespread in humans than previously appreciated and enable limited assignment of parental allelic transmissions in unrelated individuals. bioRxiv. 10.1101/161471

[B21] Cunningham F. Amode M. R. Barrell D. Beal K. Billis K. Brent S.. (2015). Ensembl 2015. Nucleic Acids Res. 43, D662–D669. 10.1093/nar/gku1010 25352552 PMC4383879

[B22] Da Silva-Santiago S. C. Pacheco C. Rocha T. C. Brasil S. M. Pacheco A. C. Silva M. M.. (2014). The linked human imprintome v1.0: over 120 genes confirmed as imprinted impose a major review on previous censuses. Int. J. Data Min. Bioinform. 10, 329–356. 10.1504/IJDMB.2014.064547 25946867

[B23] Do C. Lang C. F. Lin J. Darbary H. Krupska I. Gaba A.. (2016). Mechanisms and disease associations of haplotype-dependent allele-specific DNA methylation. Am. J. Hum. Genet. 98, 934–955. 10.1016/j.ajhg.2016.03.027 27153397 PMC4863666

[B24] Docherty L. E. Rezwan F. I. Poole R. L. Jagoe H. Lake H. Lockett G. A.. (2014). Genome-wide DNA methylation analysis of patients with imprinting disorders identifies differentially methylated regions associated with novel candidate imprinted genes. J. Med. Genet. 51, 229–238. 10.1136/jmedgenet-2013-102116 24501229 PMC3963529

[B25] Eggermann T. Perez De Nanclares G. Maher E. R. Temple I. K. Tumer Z. Monk D.. (2015). Imprinting disorders: a group of congenital disorders with overlapping patterns of molecular changes affecting imprinted loci. Clin. Epigenetics 7:123. 10.1186/s13148-015-0143-8 26583054 PMC4650860

[B26] Ernst J. Kellis M. (2015). Large-scale imputation of epigenomic datasets for systematic annotation of diverse human tissues. Nat. Biotechnol. 33, 364–376. 10.1038/nbt.3157 25690853 PMC4512306

[B27] Fang F. Hodges E. Molaro A. Dean M. Hannon G. J. Smith A. D. (2012). Genomic landscape of human allele-specific DNA methylation. Proc. Natl. Acad. Sci. U.S.A. 109, 7332–7337. 10.1073/pnas.1201310109 22523239 PMC3358917

[B28] Green B. B. Kappil M. Lambertini L. Armstrong D. A. Guerin D. J. Sharp A. J.. (2015). Expression of imprinted genes in placenta is associated with infant neurobehavioral development. Epigenetics 10, 834–841. 10.1080/15592294.2015.1073880 26198301 PMC4623032

[B29] Guo H. Zhu P. Yan L. Li R. Hu B. Lian Y.. (2014). The DNA methylation landscape of human early embryos. Nature 511, 606–610. 10.1038/nature13544 25079557

[B30] Hamada H. Okae H. Toh H. Chiba H. Hiura H. Shirane K.. (2016). Allele-specific methylome and transcriptome analysis reveals widespread imprinting in the human placenta. Am. J. Hum. Genet. 99, 1045–1058. 10.1016/j.ajhg.2016.08.021 27843122 PMC5097938

[B31] Hanna C. W. Peñaherrera M. S. Saadeh H. Andrews S. Mcfadden D. E. Kelsey G.. (2016). Pervasive polymorphic imprinted methylation in the human placenta. Genome Res. 26, 756–767. 10.1101/gr.196139.115 26769960 PMC4889973

[B32] Hannula-Jouppi K. Muurinen M. Lipsanen-Nyman M. Reinius L. E. Ezer S. Greco D.. (2014). Differentially methylated regions in maternal and paternal uniparental disomy for chromosome 7. Epigenetics 9, 351–365. 10.4161/epi.27160 24247273 PMC4053454

[B33] Hatt L. Aagaard M. M. Bach C. Graakjaer J. Sommer S. Agerholm I. E.. (2016). Microarray-based analysis of methylation of 1st trimester trisomic placentas from down syndrome, edwards syndrome and patau syndrome. PLoS ONE 11:e0160319. 10.1371/journal.pone.0160319 27490343 PMC4973974

[B34] Hatt L. Aagaard M. M. Graakjaer J. Bach C. Sommer S. Agerholm I. E.. (2015). Microarray-Based Analysis of Methylation Status of CpGs in Placental DNA and Maternal Blood DNA–potential new epigenetic biomarkers for cell free Fetal DNA-based diagnosis. PLoS ONE 10:e0128918. 10.1371/journal.pone.0128918 26230497 PMC4521692

[B35] Hinrichs A. S. Raney B. J. Speir M. L. Rhead B. Casper J. Karolchik D.. (2016). UCSC data integrator and variant annotation integrator. Bioinformatics 32, 1430–1432. 10.1093/bioinformatics/btv766 26740527 PMC4848401

[B36] Hitchins M. P. Monk D. Bell G. M. Ali Z. Preece M. A. Stanier P.. (2001). Maternal repression of the human GRB10 gene in the developing central nervous system; evaluation of the role for GRB10 in Silver-Russell syndrome. Eur. J. Hum. Genet. 9, 82–90. 10.1038/sj.ejhg.5200583 11313740

[B37] Jirtle J. Murphy C. (2012). Geneimprint database [Online]. Available online at: http://www.geneimprint.com (Accessed September 10, 2017).

[B38] Kagami M. Matsubara K. Nakabayashi K. Nakamura A. Sano S. Okamura K.. (2017). Genome-wide multilocus imprinting disturbance analysis in Temple syndrome and Kagami-Ogata syndrome. Genet. Med. 19, 476–482. 10.1038/gim.2016.123 27632690 PMC5392596

[B39] Karim S. Noureldin H. F. Abusamra H. Salem N. Alhathli E. Dudley J.. (2016). e-GRASP: an integrated evolutionary and GRASP resource for exploring disease associations. BMC Genomics 17:770. 10.1186/s12864-016-3088-1 27766955 PMC5073857

[B40] Kent W. J. Sugnet C. W. Furey T. S. Roskin K. M. Pringle T. H. Zahler A. M.. (2002). The human genome browser at UCSC. Genome Res. 12, 996–1006. 10.1101/gr.229102 12045153 PMC186604

[B41] Kobayashi H. Yanagisawa E. Sakashita A. Sugawara N. Kumakura S. Ogawa H.. (2013). Epigenetic and transcriptional features of the novel human imprinted lncRNA GPR1AS suggest it is a functional ortholog to mouse Zdbf2linc. Epigenetics 8, 635–645. 10.4161/epi.24887 23764515 PMC3857343

[B42] Lek M. Karczewski K. J. Minikel E. V. Samocha K. E. Banks E. Fennell T.. (2016). Analysis of protein-coding genetic variation in 60,706 humans. Nature 536, 285–291. 10.1038/nature19057 27535533 PMC5018207

[B43] Li E. Zhang Y. (2014). DNA methylation in mammals. Cold Spring Harb. Perspect. Biol. 6:a019133. 10.1101/cshperspect.a019133 24789823 PMC3996472

[B44] Lizio M. Harshbarger J. Abugessaisa I. Noguchi S. Kondo A. Severin J.. (2017). Update of the FANTOM web resource: high resolution transcriptome of diverse cell types in mammals. Nucleic Acids Res. 45, D737–D743. 10.1093/nar/gkw995 27794045 PMC5210666

[B45] Luedi P. P. Dietrich F. S. Weidman J. R. Bosko J. M. Jirtle R. L. Hartemink A. J. (2007). Computational and experimental identification of novel human imprinted genes. Genome Res. 17, 1723–1730. 10.1101/gr.6584707 18055845 PMC2099581

[B46] Machado F. B. Machado F. B. Faria M. A. Lovatel V. L. Alves Da Silva A. F. Radic C. P.. (2014). 5meCpG epigenetic marks neighboring a primate-conserved core promoter short tandem repeat indicate X-chromosome inactivation. PLoS ONE 9:e103714. 10.1371/journal.pone.0103714 25078280 PMC4117532

[B47] Maeda T. Higashimoto K. Jozaki K. Yatsuki H. Nakabayashi K. Makita Y.. (2014). Comprehensive and quantitative multilocus methylation analysis reveals the susceptibility of specific imprinted differentially methylated regions to aberrant methylation in Beckwith-Wiedemann syndrome with epimutations. Genet. Med. 16, 903–912. 10.1038/gim.2014.46 24810686 PMC4262761

[B48] Marzi S. J. Meaburn E. L. Dempster E. L. Lunnon K. Paya-Cano J. L. Smith R. G.. (2016). Tissue-specific patterns of allelically-skewed DNA methylation. Epigenetics 11, 24–35. 10.1080/15592294.2015.1127479 26786711 PMC4846124

[B49] McCann J. A. Zheng H. Islam A. Goodyer C. G. Polychronakos C. (2001). Evidence against GRB10 as the gene responsible for Silver-Russell syndrome. Biochem. Biophys. Res. Commun. 286, 943–948. 10.1006/bbrc.2001.5500 11527390

[B50] Mergenthaler S. Hitchins M. P. Blagitko-Dorfs N. Monk D. Wollmann H. A. Ranke M. B.. (2001). Conflicting reports of imprinting status of human GRB10 in developing brain: how reliable are somatic cell hybrids for predicting allelic origin of expression? Am. J. Hum. Genet. 68, 543–545. 10.1086/318192 11170901 PMC1235290

[B51] Molaro A. Hodges E. Fang F. Song Q. Mccombie W. R. Hannon G. J.. (2011). Sperm methylation profiles reveal features of epigenetic inheritance and evolution in primates. Cell 146, 1029–1041. 10.1016/j.cell.2011.08.016 21925323 PMC3205962

[B52] Monahan K. Lomvardas S. (2015). Monoallelic expression of olfactory receptors. Annu. Rev. Cell Dev. Biol. 31, 721–740. 10.1146/annurev-cellbio-100814-125308 26359778 PMC4882762

[B53] Monk D. Arnaud P. Frost J. Hills F. A. Stanier P. Feil R.. (2009). Reciprocal imprinting of human GRB10 in placental trophoblast and brain: evolutionary conservation of reversed allelic expression. Hum. Mol. Genet. 18, 3066–3074. 10.1093/hmg/ddp248 19487367

[B54] Morison I. M. Ramsay J. P. Spencer H. G. (2005). A census of mammalian imprinting. Trends Genet. 21, 457–465. 10.1016/j.tig.2005.06.008 15990197

[B55] Narasimhan V. M. Hunt K. A. Mason D. Baker C. L. Karczewski K. J. Barnes M. R.. (2016). Health and population effects of rare gene knockouts in adult humans with related parents. Science 352, 474–477. 10.1126/science.aac8624 26940866 PMC4985238

[B56] NCBI Resource Coordinators (2017). Database Resources of the National Center for Biotechnology Information. Nucleic Acids Res. 45, D12–D17. 10.1093/nar/gkw1071 27899561 PMC5210554

[B57] NHLBI GO Exome Sequencing Project (2012). Exome Variant Server [Online]. Seattle, WA Available online: http://evs.gs.washington.edu/EVS/ (Accessed April 29, 2016).

[B58] Okae H. Chiba H. Hiura H. Hamada H. Sato A. Utsunomiya T.. (2014). Genome-wide analysis of DNA methylation dynamics during early human development. PLoS Genet. 10:e1004868. 10.1371/journal.pgen.1004868 25501653 PMC4263407

[B59] Paliwal A. Temkin A. M. Kerkel K. Yale A. Yotova I. Drost N.. (2013). Comparative anatomy of chromosomal domains with imprinted and non-imprinted allele-specific DNA methylation. PLoS Genet. 9:e1003622. 10.1371/journal.pgen.1003622 24009515 PMC3757050

[B60] Poot M. Kroes H. Y. Se V. D. W. Eleveld M. J. Rooms L. Nievelstein R. A.. (2007). Dandy-Walker complex in a boy with a 5 Mb deletion of region 1q44 due to a paternal t(1;20)(q44;q13.33). Am. J. Med. Genet. A 143A, 1038–1044. 10.1002/ajmg.a.31690 17431901

[B61] Ramos E. M. Hoffman D. Junkins H. A. Maglott D. Phan L. Sherry S. T.. (2014). Phenotype-Genotype Integrator (PheGenI): synthesizing genome-wide association study (GWAS) data with existing genomic resources. Eur. J. Hum. Genet. 22, 144–147. 10.1038/ejhg.2013.96 23695286 PMC3865418

[B62] Raney B. J. Dreszer T. R. Barber G. P. Clawson H. Fujita P. A. Wang T.. (2014). Track data hubs enable visualization of user-defined genome-wide annotations on the UCSC Genome Browser. Bioinformatics 30, 1003–1005. 10.1093/bioinformatics/btt637 24227676 PMC3967101

[B63] Roadmap Epigenomics Consortium Kundaje A. Meuleman W. Ernst J. Bilenky M. Yen A. Heravi-Moussavi A.. (2015). Integrative analysis of 111 reference human epigenomes. Nature 518, 317–330. 10.1038/nature14248 25693563 PMC4530010

[B64] Rochtus A. Martin-Trujillo A. Izzi B. Elli F. Garin I. Linglart A.. (2016). Genome-wide DNA methylation analysis of pseudohypoparathyroidism patients with GNAS imprinting defects. Clin. Epigenetics 8:10. 10.1186/s13148-016-0175-8 26819647 PMC4728790

[B65] Rodrigues J. A. Zilberman D. (2015). Evolution and function of genomic imprinting in plants. Genes Dev. 29, 2517–2531. 10.1101/gad.269902.115 26680300 PMC4699382

[B66] Rodriguez J. M. Maietta P. Ezkurdia I. Pietrelli A. Wesselink J. J. Lopez G.. (2013). APPRIS: annotation of principal and alternative splice isoforms. Nucleic Acids Res. 41, D110–D117. 10.1093/nar/gks1058 23161672 PMC3531113

[B67] Romanelli V. Nakabayashi K. Vizoso M. Moran S. Iglesias-Platas I. Sugahara N.. (2014). Variable maternal methylation overlapping the nc886/vtRNA2-1 locus is locked between hypermethylated repeats and is frequently altered in cancer. Epigenetics 9, 783–790. 10.4161/epi.28323 24589629 PMC4063837

[B68] Sanchez-Delgado M. Court F. Vidal E. Medrano J. Monteagudo-Sanchez A. Martin-Trujillo A.. (2016a). Human Oocyte-Derived Methylation Differences Persist in the Placenta Revealing Widespread Transient Imprinting. PLoS Genet. 12:e1006427. 10.1371/journal.pgen.1006427 27835649 PMC5106035

[B69] Sanchez-Delgado M. Riccio A. Eggermann T. Maher E. R. Lapunzina P. Mackay D.. (2016b). Causes and Consequences of Multi-Locus Imprinting Disturbances in Humans. Trends Genet. 32, 444–455. 10.1016/j.tig.2016.05.001 27235113

[B70] Savol A. J. Wang P. I. Jeon Y. Colognori D. Yildirim E. Pinter S. F.. (2017). Genome-wide identification of autosomal genes with allelic imbalance of chromatin state. PLoS ONE 12:e0182568. 10.1371/journal.pone.0182568 28796844 PMC5552117

[B71] Severin J. Lizio M. Harshbarger J. Kawaji H. Daub C. O. Hayashizaki Y.. (2014). Interactive visualization and analysis of large-scale sequencing datasets using ZENBU. Nat. Biotechnol. 32, 217–219. 10.1038/nbt.2840 24727769

[B72] Sherry S. T. Ward M. H. Kholodov M. Baker J. Phan L. Smigielski E. M.. (2001). dbSNP: the NCBI database of genetic variation. Nucleic Acids Res. 29, 308–311. 10.1093/nar/29.1.308 11125122 PMC29783

[B73] Skaar D. A. Li Y. Bernal A. J. Hoyo C. Murphy S. K. Jirtle R. L. (2012). The human imprintome: regulatory mechanisms, methods of ascertainment, and roles in disease susceptibility. ILAR J. 53, 341–358. 10.1093/ilar.53.3-4.341 23744971 PMC3683658

[B74] Smith Z. D. Chan M. M. Humm K. C. Karnik R. Mekhoubad S. Regev A.. (2014). DNA methylation dynamics of the human preimplantation embryo. Nature 511, 611–615. 10.1038/nature13581 25079558 PMC4178976

[B75] Soellner L. Begemann M. Mackay D. J. Gronskov K. Tumer Z. Maher E. R.. (2017). Recent advances in imprinting disorders. Clin. Genet. 91, 3–13. 10.1111/cge.12827 27363536

[B76] Song Q. Decato B. Hong E. E. Zhou M. Fang F. Qu J.. (2013). A reference methylome database and analysis pipeline to facilitate integrative and comparative epigenomics. PLoS ONE 8:e81148. 10.1371/journal.pone.0081148 24324667 PMC3855694

[B77] Staley J. R. Blackshaw J. Kamat M. A. Ellis S. Surendran P. Sun B. B.. (2016). PhenoScanner: a database of human genotype-phenotype associations. Bioinformatics 32, 3207–3209. 10.1093/bioinformatics/btw373 27318201 PMC5048068

[B78] Sudmant P. H. Rausch T. Gardner E. J. Handsaker R. E. Abyzov A. Huddleston J.. (2015). An integrated map of structural variation in 2,504 human genomes. Nature 526, 75–81. 10.1038/nature15394 26432246 PMC4617611

[B79] Sulem P. Helgason H. Oddson A. Stefansson H. Gudjonsson S. A. Zink F.. (2015). Identification of a large set of rare complete human knockouts. Nat. Genet. 47, 448–452. 10.1038/ng.3243 25807282

[B80] The GTEx Project C. (2015). Human genomics. The Genotype-Tissue Expression (GTEx) pilot analysis: multitissue gene regulation in humans. Science 348, 648–660. 10.1126/science.1262110 25954001 PMC4547484

[B81] van Dongen J. Nivard M. G. Baselmans B. M. Zilhao N. R. Ligthart L. Consortium B.. (2015). Epigenome-wide association study of aggressive behavior. Twin Res. Hum. Genet. 18, 686–698. 10.1017/thg.2015.74 26508088

[B82] van Kempen L. C. Redpath M. Elchebly M. Klein K. O. Papadakis A. I. Wilmott J. S.. (2016). The protein phosphatase 2A regulatory subunit PR70 is a gonosomal melanoma tumor suppressor gene. Sci. Transl. Med. 8, 369ra177. 10.1126/scitranslmed.aai9188 27974665

[B83] Viana J. Pidsley R. Troakes C. Spiers H. Wong C. C. Al-Sarraj S.. (2014). Epigenomic and transcriptomic signatures of a Klinefelter syndrome (47,XXY) karyotype in the brain. Epigenetics 9, 587–599. 10.4161/epi.27806 24476718 PMC4121369

[B84] Wan E. S. Qiu W. Morrow J. Beaty T. H. Hetmanski J. Make B. J.. (2015). Genome-wide site-specific differential methylation in the blood of individuals with Klinefelter syndrome. Mol. Reprod. Dev. 82, 377–386. 10.1002/mrd.22483 25988574 PMC4439255

[B85] Ward L. D. Kellis M. (2016). HaploReg v4: systematic mining of putative causal variants, cell types, regulators and target genes for human complex traits and disease. Nucleic Acids Res. 44, D877–D881. 10.1093/nar/gkv1340 26657631 PMC4702929

[B86] Wei Y. Su J. Liu H. Lv J. Wang F. Yan H.. (2014). MetaImprint: an information repository of mammalian imprinted genes. Development 141, 2516–2523. 10.1242/dev.105320 24850854

[B87] Yoshihashi H. Maeyama K. Kosaki R. Ogata T. Tsukahara M. Goto Y.. (2000). Imprinting of human GRB10 and its mutations in two patients with Russell-Silver syndrome. Am. J. Hum. Genet. 67, 476–482. 10.1086/302997 10856193 PMC1287191

[B88] Yuen R. K. Avila L. Penaherrera M. S. Von Dadelszen P. Lefebvre L. Kobor M. S.. (2009). Human placental-specific epipolymorphism and its association with adverse pregnancy outcomes. PLoS ONE 4:e7389. 10.1371/journal.pone.0007389 19838307 PMC2760756

[B89] Yuen R. K. Jiang R. Penaherrera M. S. Mcfadden D. E. Robinson W. P. (2011). Genome-wide mapping of imprinted differentially methylated regions by DNA methylation profiling of human placentas from triploidies. Epigenetics Chromatin 4:10. 10.1186/1756-8935-4-10 21749726 PMC3154142

[B90] Zhang Y. Guan D. G. Yang J. H. Shao P. Zhou H. Qu L. H. (2010). ncRNAimprint: a comprehensive database of mammalian imprinted noncoding RNAs. RNA 16, 1889–1901. 10.1261/rna.2226910 20801769 PMC2941098

[B91] Zhou X. Maricque B. Xie M. Li D. Sundaram V. Martin E. A.. (2011). The human epigenome browser at Washington University. Nat. Methods 8, 989–990. 10.1038/nmeth.1772 22127213 PMC3552640

