# Supplementary figures and images for: Maternal 5mCpG Imprints at the PARD6G-AS1 and GCSAML Differentially Methylated Regions Are Decoupled From Parent-of-Origin Expression Effects in Multiple Human Tissues
Source: Front Genet. 2018 Mar 1;9:36. doi: 10.3389/fgene.2018.00036 (PMC5838017; doi:10.3389/fgene.2018.00036)

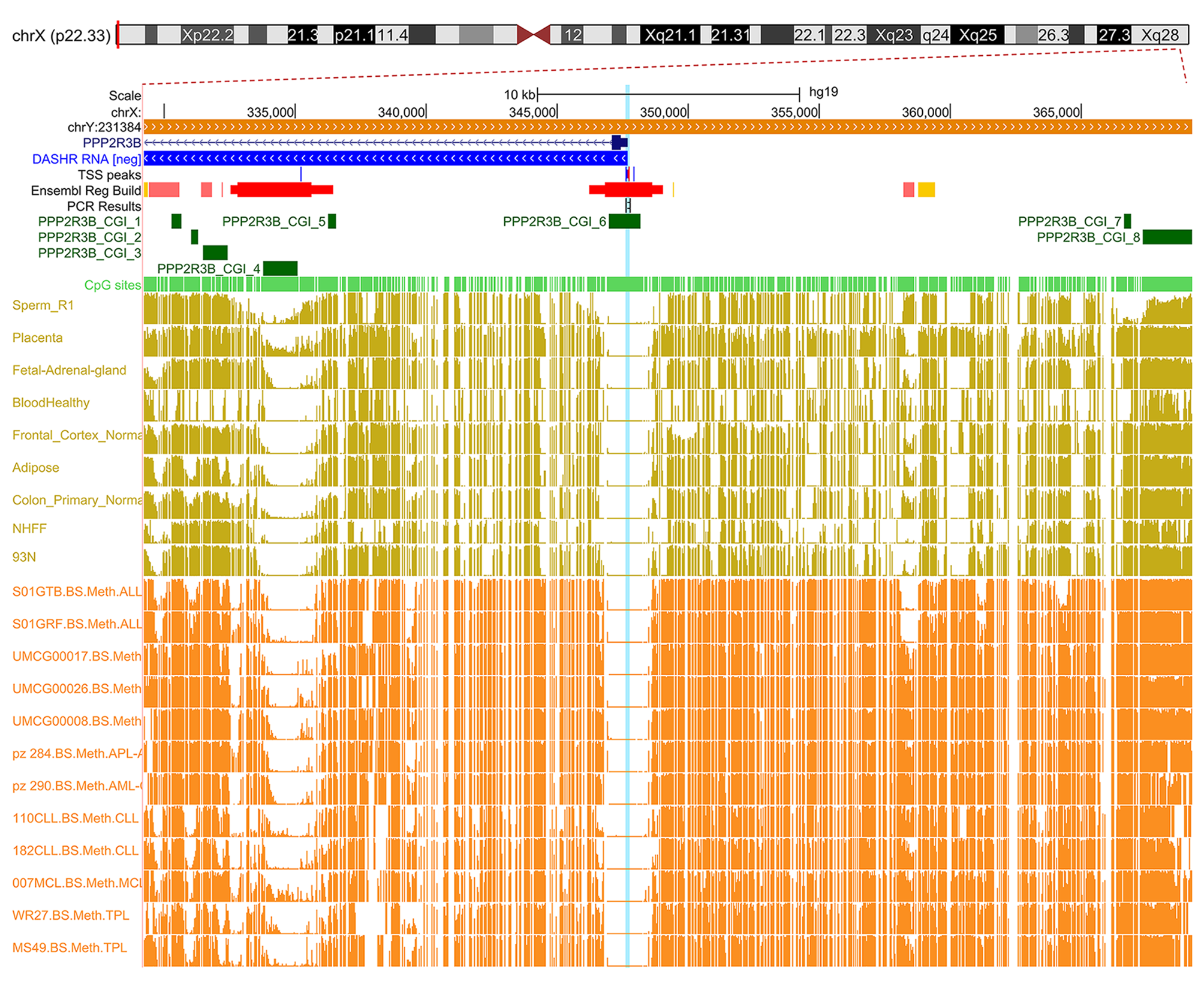

Supplement: Figure S1 — Constitutive unmethylated status at the PPP2R3B predicted promoter region. X- chromosome ideogram; physical positions and domain features of the PPP2R3B locus depicting the methylation status at CpG sites across a 40 kb long-span view (hg19; 329240–369239). The methylation levels are represented on a scale from 0 to 1 (hypomethylated to hypermethylated). The image is centered on CGI-6, localized in the PPP2R3B predicted promoter region. The light green ticks represent the positions of the CpG sites. The PPP2R3B gene is transcribed from the minus DNA strand. The annotated features are (from top to bottom) the segmental duplication in chrY (chrY:231384 track), the exon-intron organization of the principal isoform, the evidence of small non-coding RNAs (sncRNAs) (DASHR RNA [neg] track), the FANTOM5 transcriptional start sites (TSS peaks track) (Lizio et al., 2017), the Ensembl Regulatory Build predicted promoters (Ensembl Reg Build track) (Cunningham et al., 2015), CpG islands and CGI-bearing AMRs (this study) and the comparative custom Fang_AMR track (Fang et al., 2012). A constitutive unmethylated status is observed across PPP2R3B CGI-6 in gametes, as well as in healthy (golden ticks) and cancerous (orange ticks) adult tissues. The chromosomal position of the region corresponding to the HpaII-sensitive amplimer (PCR results track) is highlighted in light blue within PPP2R3B CGI-6. [file Image1.TIF]

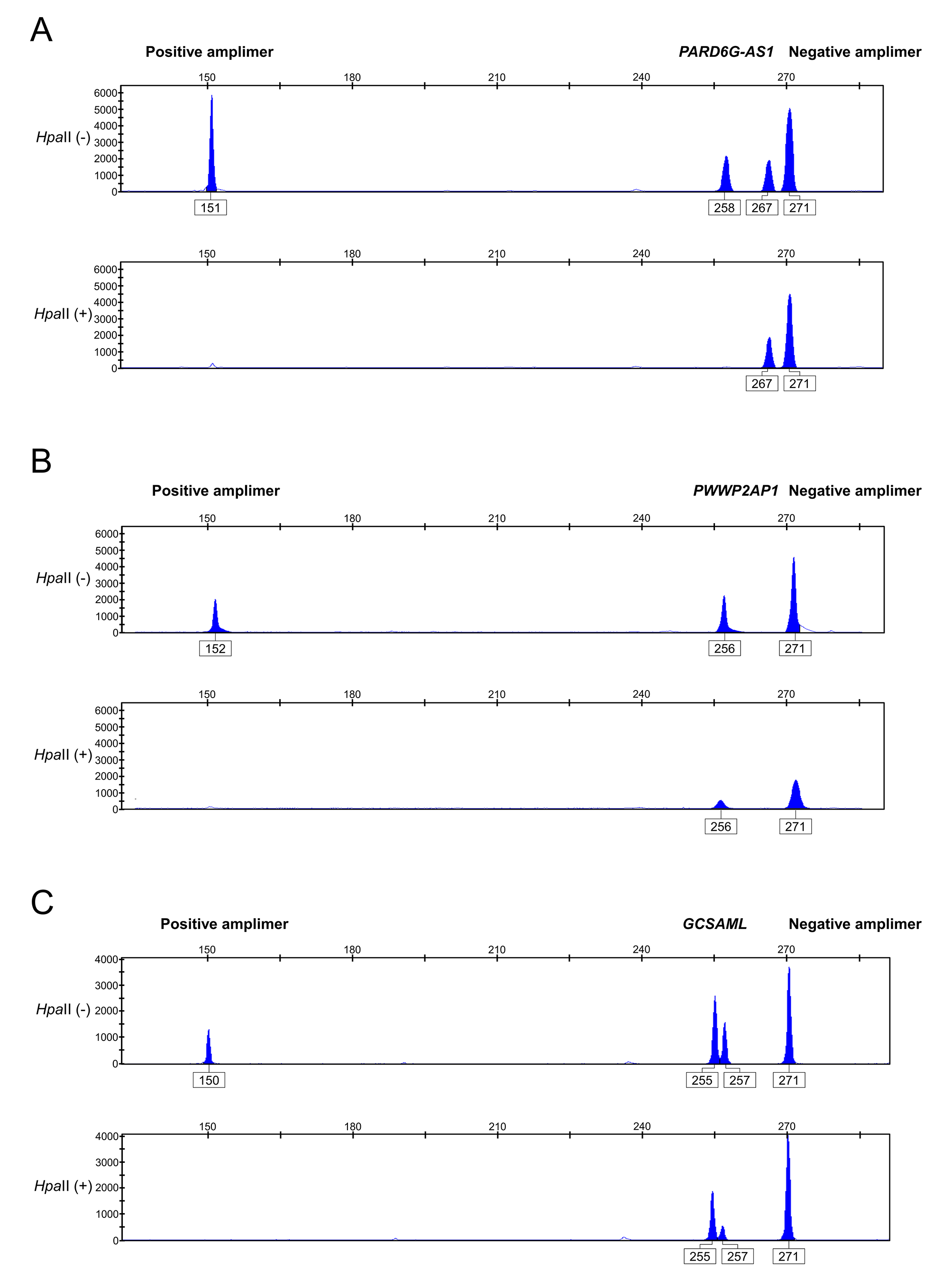

Supplement: Figure S2 — Validation of the intermediate methylation statuses at the PARD6G-AS1, PWWP2AP1, and GCSAML DMRs by MSRE-PCR triplex assays. Representative methylation profiles before and after digestion with the methylation-sensitive HpaII restriction enzyme. (A) PARD6G-AS1 DMR, (B) PWWP2AP1 DMR, and (C) GCSAML DMR. Each profile generated from uncut DNA consists of two control products (left and right peaks) and a test product (peak in the middle). The control products correspond to the amplimers of a chromosomal region known to be 100% unmethylated in the human genome (left peak) (see Materials and Methods for details) and a chromosomal region bearing no HpaII sites (right peak). The PARD6G-AS1 DMR amplimer comprises the common indel variant rs11281142 (MAF > 0.4), and thus, a heterozygous sample yields two peaks representing the two variant alleles, one with and the other without the CTGTGGTGC insertion. The GCSAML DMR amplimer comprises two peaks (255 and 257 bp), consistent with the addition of a 3′-end A residue overhang to the PCR product, since the two-peak product occurs in 20 genomic DNA samples tested, and the two annotated, encompassing, one-base-pair indel variants rs533592199 (-/G), and rs773445240 (-/T) reported in dbSNP150 have not been detected in over 841,883 public exomes (NHLBI GO Exome Sequencing Project, 2012; Sulem et al., 2015; Chen et al., 2016; Lek et al., 2016; Narasimhan et al., 2016). Each panel corresponds to a different individual from one out of three representative nuclear families that were informative for at least one SNP. The biallelic methylation profile at the PWWP2AP1 hemimethylated CGI DMR was confirmed using a second MSRE (HhaI) in two informative individuals (data not shown). [file Image2.TIF]

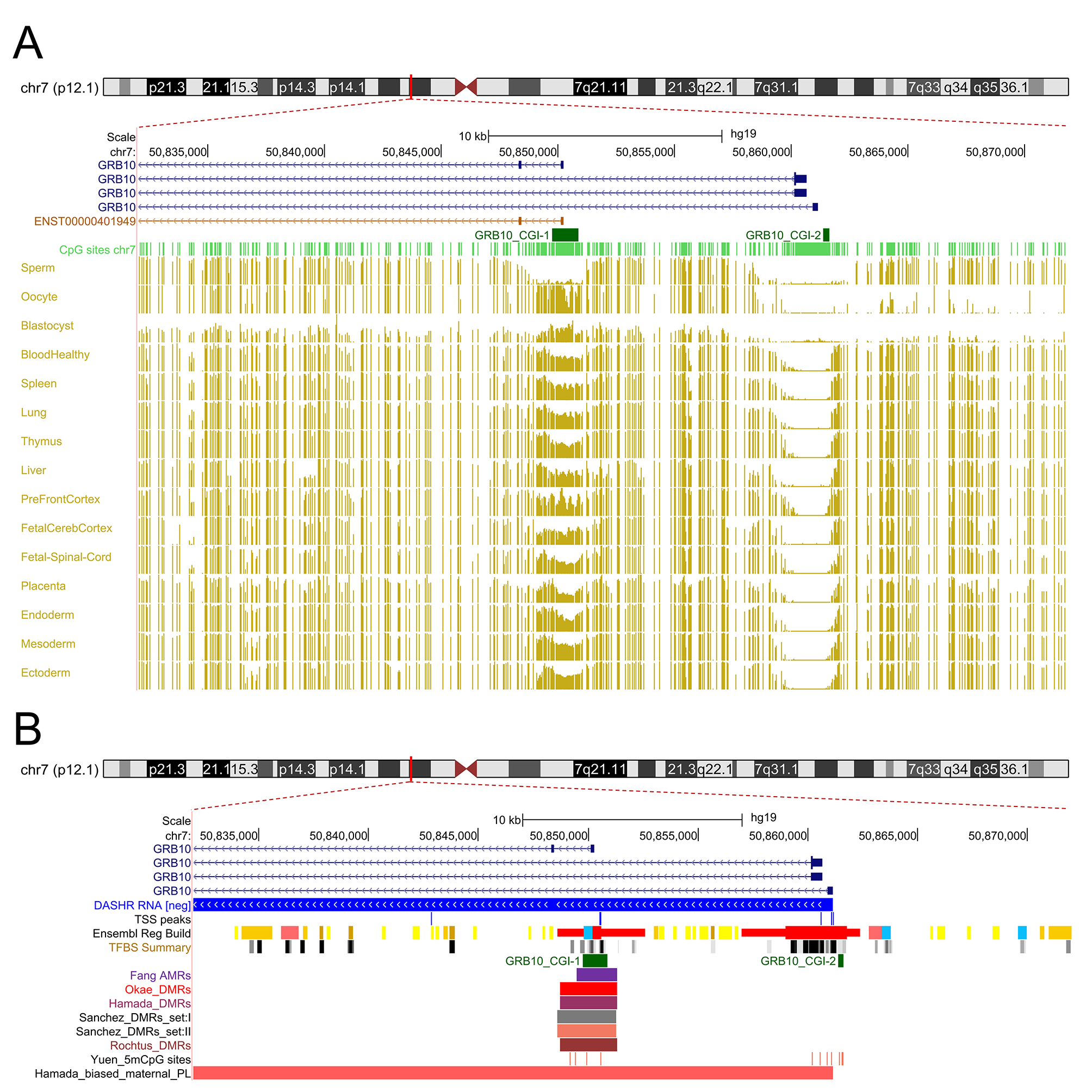

Supplement: Figure S3 — Constitutive hemimethylation statuses across the known GRB10 maternal iDMR. (A) Chromosome 7 ideogram; physical positions and domain features of the GRB10 locus showing the methylation status at CpG sites (golden ticks) across a 40 kb long-span view (hg19; chr7:50832014-50872013). The image is centered on the CGI localized in the GRB10 predicted promoter region, labeled “GRB10 maternal iDMR” (GRB10 CGI-1), which maps to a predicted secondary promoter region upstream of the transcriptional start site of the short GRB10 isoform. The GRB10 locus is transcribed from the minus DNA strand. The light green ticks represent the positions of the CpG sites. The methylation levels are represented on a scale from 0 to 1 (hypomethylated to hypermethylated). The methylation levels across the CpG sites within the GRB10 maternal iDMR range from 0.35 to 0.65 in adult somatic tissues. In the same region, however, there is asymmetrical methylation in gametes (hypermethylation in oocytes and hypomethylation in spermatozoa). (B) Cross-reference overlaps of the GRB10 maternal iDMR with differentially methylated regions reported in control-disease methylomes and healthy placentas. The annotated features are (from top to bottom) the exon-intron organization of the principal and alternative isoforms, the evidence of large intergenic non-coding RNAs (lincRNA) and small non-coding RNAs (sncRNAs) (DASHR RNA [neg] track), the FANTOM5 transcriptional start sites (TSS peaks track) (Lizio et al., 2017), the Ensembl Regulatory Build predicted promoters (Ensembl Reg Build track) and transcriptional factor binding sites (TFBS summary track) (Cunningham et al., 2015), CpG islands and the comparative custom Fang_AMR track (Fang et al., 2012). The GRB10 maternal iDMR overlaps the domain known be differentially methylated in a maternal-origin-dependent manner in blastocysts and the placenta (Okae et al., 2014; Hamada et al., 2016; Sanchez-Delgado et al., 2016a) (Okae_DMRs, Hamada_DMRs, Sanchez_DMRs_set:I [file Image3.TIF]

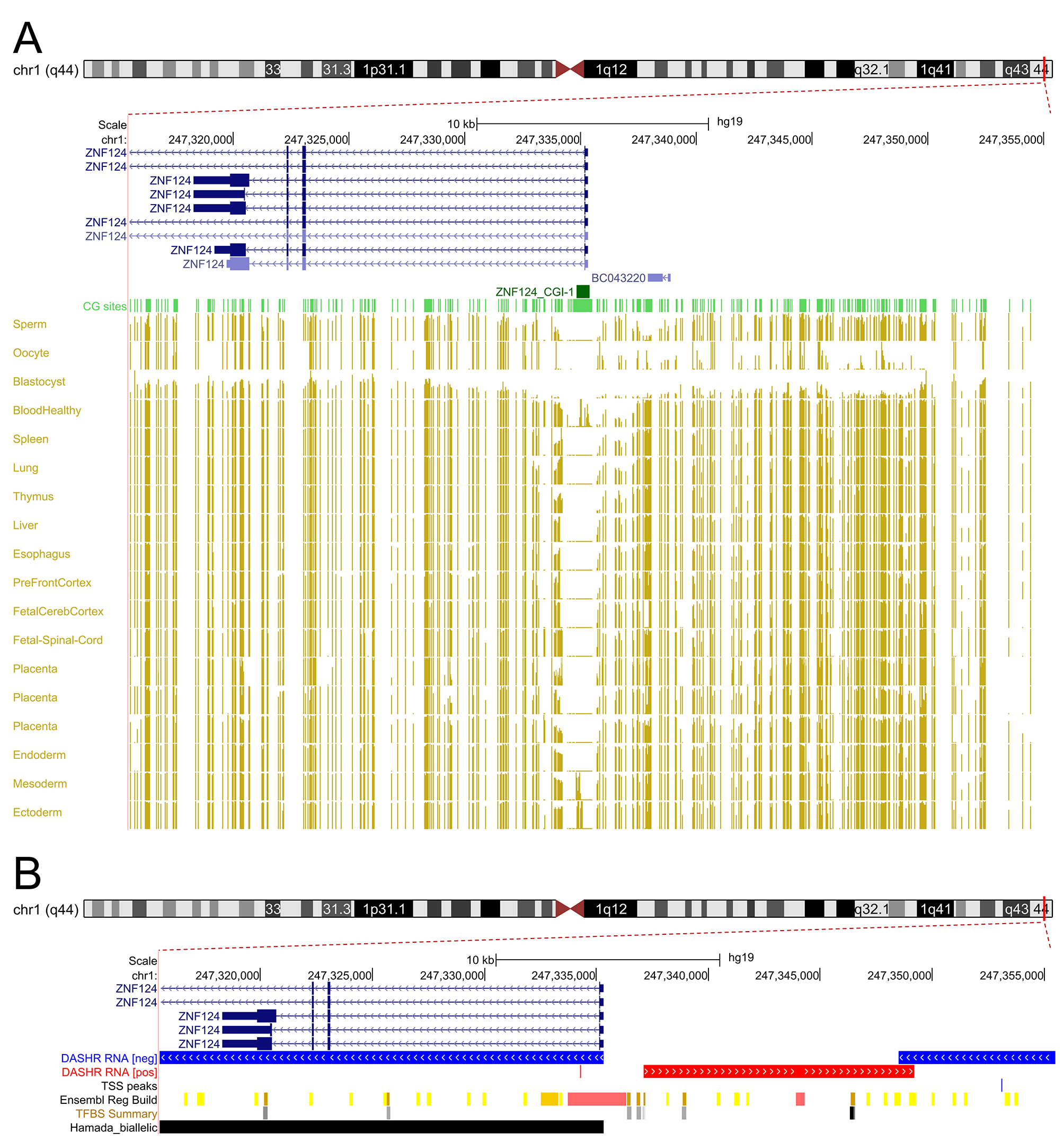

Supplement: Figure S4 — The ZNF124 locus has no overlapping hemimethylated CGIs. (A) Chromosome 1 ideogram; physical positions and domain features of the ZNF124 locus showing the methylation status at CpG sites (golden ticks) across a 40 kb long-span view (hg19; chr1:247315500–247355499). The image is centered on the CGI located in the ZNF124 predicted promoter region. The ZNF124 locus is transcribed from the minus DNA strand. The light green ticks represent the positions of the CpG sites. The ZNF124 CGI is hypomethylated in all shown BS-Seq methylomes. (B) Cross-reference overlaps of the predicted differentially methylated regions reported in healthy placentas. The annotated features are as in Figure S3. In the placenta, there is evidence of biallelic expression (Hamada et al., 2016). [file Image4.TIF]

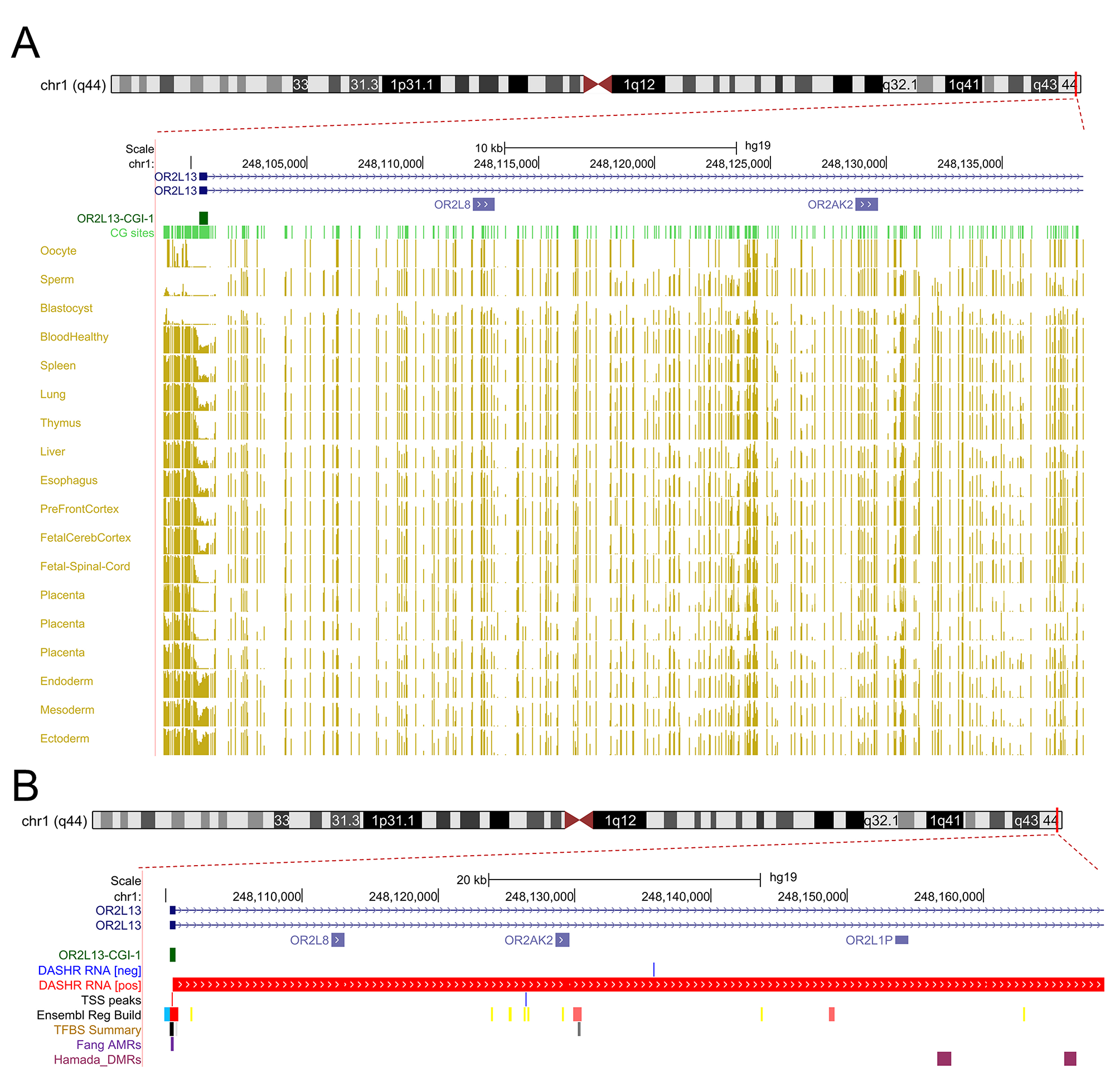

Supplement: Figure S5 — The OR2L13 locus has no overlapping hemimethylated CGIs. (A) Chromosome 1 ideogram; physical positions and domain features of the OR2L13 locus showing the methylation status at the CpG sites (golden ticks) across a 40 kb long-span view (hg19; chr1:248098501–248138500). The light green ticks represent the positions of the CpG sites. The OR2L13 CGI, localized in the OR2L13 predicted promoter region, is hypomethylated in all shown BS-Seq methylomes analyzed (average level of 0.24). (B) Cross-reference overlaps of the predicted DMR reported in healthy placentas across a 70kb long-span view (hg19; chr1:248098401–248168400). In the placenta (Hamada-DMR track), there are two intergenic predicted maternal DMRs 56.3 and 65.6 kb downstream, respectively, of the OR2L13 CGI. The annotated features are as in Figure S3. [file Image5.TIF]

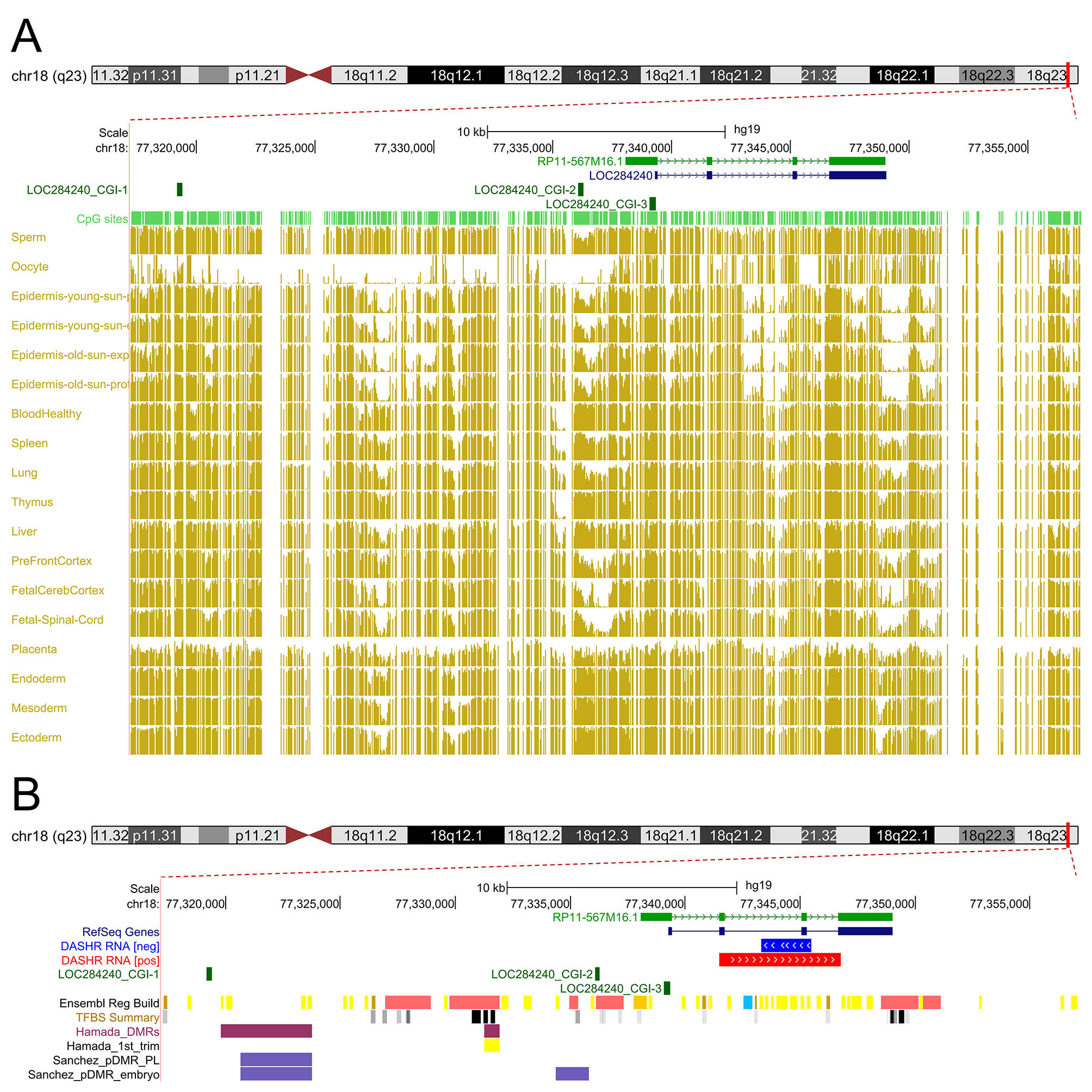

Supplement: Figure S6 — The RP11-567M16.1-LOC284240 locus exhibits a hemimethylated CGI. (A) Chromosome 18 ideogram; physical positions and domain features of the RP11-567M16.1-LOC284240 locus showing the methylation status at the CpG sites (golden ticks) across a 40 kb long-span view (hg19; chr18:77317230–77357229). The image is centered at the RP11-567M16.1-LOC284240 CGI-2 predicted promoter region. The light green ticks represent the position of the CpG sites. RP11-567M16.1-LOC284240 CGI-2 is hemimethylated (average level of 0.53) in several BS-Seq methylomes, including those of the epidermis, esophagus, lung, fetal brain cortex, fetal spinal cord, and placenta tissues, with paternal rather than maternal methylation asymmetry in gametes (this study). The occurrence of a paternal primary candidate iDMR is consistent with the observation of monoallelic expression of the locus in the pituitary (this study). (B) Cross-reference for two predicted paternal DMRs (Hamada_DMRs, hamada_1st_trim, Sanchez_pDMR_PL, and Sanchez_pDMR_embryo tracks), none of which exhibit either a constitutive or a tissue-specific hemimethylation profile. [file Image6.TIF]

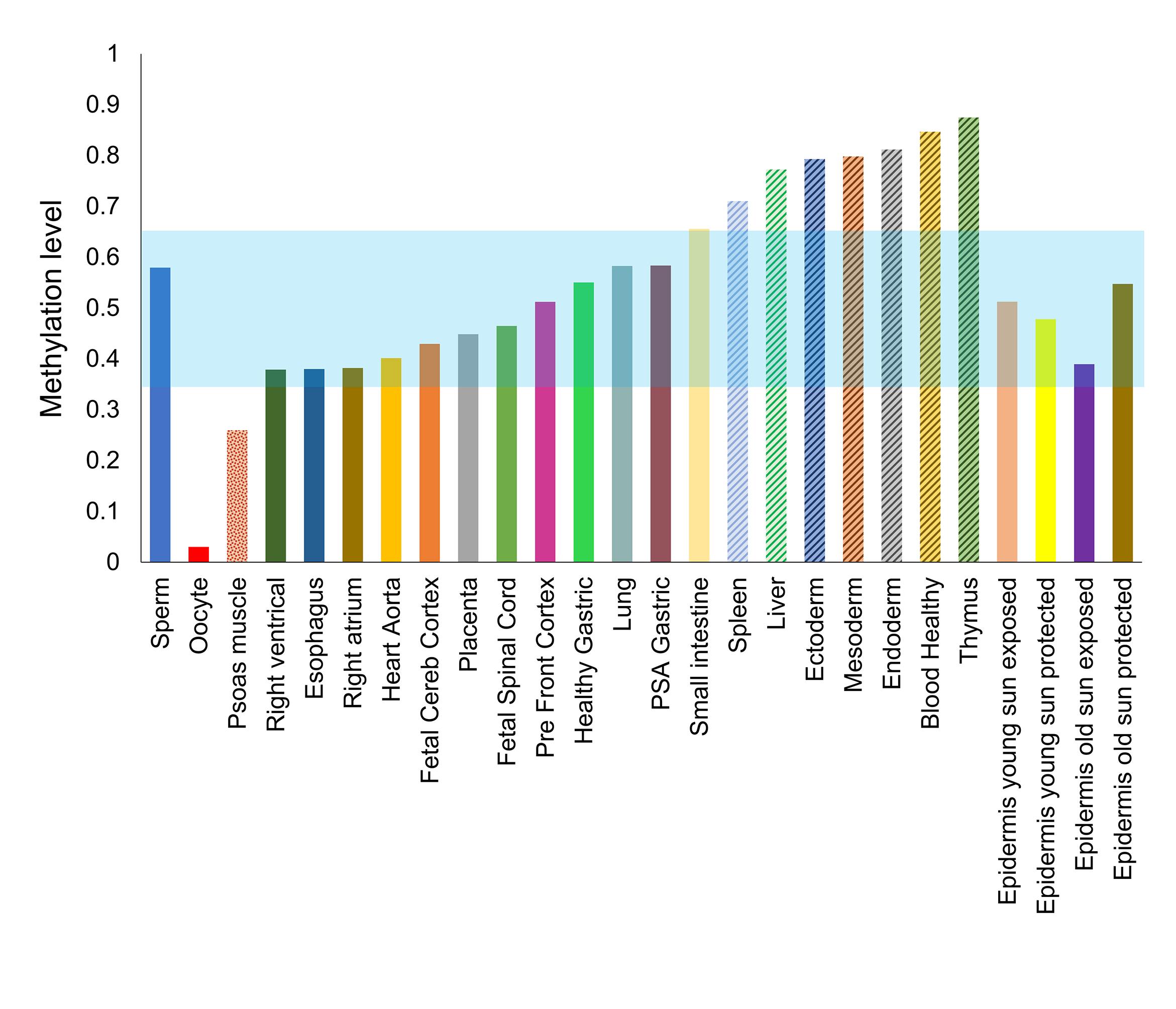

Supplement: Figure S7 — Predicted paternally-derived intermediate methylation at LOC284240 CGI-2. Methylation levels across CGI-2 at the predicted promoter-flanking region of the LOC284240 locus. Note the paternally-derived 5mCpG asymmetry in gametes and the intermediate methylation levels in BS-Seq methylomes from epidermis, lung, esophagus, gastric muscle, brain cortex, fetal spinal cord, and placenta. The hemimethylated status is not constitutive, since, in spleen, liver, blood, thymus, and the hESC-derived endodermal, mesodermal and endodermal cell lines, there is a bias toward hypermethylation. [file Image7.TIF]
